# Brain region-specific enhancement of remyelination and prevention of demyelination by the CSF1R kinase inhibitor BLZ945

**DOI:** 10.1186/s40478-018-0510-8

**Published:** 2018-02-15

**Authors:** Nicolau Beckmann, Elisa Giorgetti, Anna Neuhaus, Stefan Zurbruegg, Nathalie Accart, Paul Smith, Julien Perdoux, Ludovic Perrot, Mark Nash, Sandrine Desrayaud, Peter Wipfli, Wilfried Frieauff, Derya R. Shimshek

**Affiliations:** 1 Musculoskeletal Diseases Area, Novartis Institutes for BioMedical Research, 4002 Basel, Switzerland; 2 Neuroscience, Novartis Institutes for BioMedical Research, 4002 Basel, Switzerland; 3 Autoimmunity, Transplantation and Inflammation, Novartis Institutes for BioMedical Research, 4002 Basel, Switzerland; 4 Global Scientific Operations, Novartis Institutes for BioMedical Research, 4002 Basel, Switzerland; 5 PK Sciences, Novartis Institutes for BioMedical Research, 4002 Basel, Switzerland; 6 Preclinical Safety, Novartis Institutes for BioMedical Research, 4002 Basel, Switzerland; 7 Present address: Incyte, 1801 Augustine Cut-off, Wilmington, DE 19803 USA

**Keywords:** Neuroinflammation, Microglia, Astrocyte, Cuprizone, Myelination, Multiple sclerosis, Oligodendrocyte, CSF1R kinase, BLZ945, TREM2

## Abstract

Multiple sclerosis (MS) is a chronic inflammatory disease affecting the central nervous system (CNS). While multiple effective immunomodulatory therapies for MS exist today, they lack the scope of promoting CNS repair, in particular remyelination. Microglia play a pivotal role in regulating myelination processes, and the colony-stimulating factor 1 (CSF-1) pathway is a key regulator for microglia differentiation and survival. Here, we investigated the effects of the CSF-1 receptor kinase inhibitor, BLZ945, on central myelination processes in the 5-week murine cuprizone model by non-invasive and longitudinal magnetic resonance imaging (MRI) and histology. Therapeutic 2-week BLZ945 treatment caused a brain region-specific enhancement of remyelination in the striatum/cortex, which was absent in the corpus callosum/external capsule. This beneficial effect correlated positively with microglia reduction, increased oligodendrocytes and astrogliosis. Prophylactic BLZ945 treatment prevented excessive demyelination in the corpus callosum by reducing microglia and increasing oligondendrocytes. In the external capsule oligodendrocytes were depleted but not microglia and a buildup of myelin debris and axonal damage was observed. A similar microglial dysfunction in the external capsule with an increase of myelin debris was obvious in triggering receptor expressed on myeloid cells 2 (TREM2) knock-out mice treated with cuprizone. Finally, therapeutic BLZ945 treatment did not change the disease course in experimental autoimmune encephalomyelitis mice, a peripherally driven neuroinflammation model. Taken together, our data suggest that a short-term therapeutic inhibition of the CSF-1 receptor pathway by BLZ945 in the murine cuprizone model enhances central remyelination by modulating neuroinflammation. Thus, microglia-modulating therapies could be considered clinically for promoting myelination in combination with standard-of-care treatments in MS patients.

**Electronic supplementary material:**

The online version of this article (10.1186/s40478-018-0510-8) contains supplementary material, which is available to authorized users.

## Introduction

Multiple sclerosis (MS) is a chronic immune-mediated multifocal demyelinating disease of the central nervous system (CNS) with progressive neurodegeneration. The pathology is believed to be mainly driven by the adaptive immune-system and/or autoimmune mechanisms that damage neurons and oligodendrocytes (ODs) in the CNS leading to white and grey matter degeneration. The relevance of the innate immunity of the CNS in disease initiation and progression has not been extensively investigated. Activated microglia and astrocyte together with neurodegeneration has been observed in MS patients and this neuroinflammatory condition is believed to play an important role in the grey matter damage (for review see [3, 35]). Although white matter lesions are the classical hallmarks of MS, the demyelinating events observed in the grey matter might have a more profound contribution to the permanent neurological dysfunction than assumed. Available treatments for MS primarily address the peripheral inflammation and help relapsing-remitting MS patients to gain normal neurological function again. However, a substantial number of MS patients suffer from irreversible progression of clinical disability. The progressive phase of MS is believed to be the consequence of altered central function of microglia, astrocytes and ODs. In this neuroinflammatory milieu ODs and oligodendrocyte precursor cells (OPCs) seem not to be able to exert their proper function anymore, mainly remyelination, and thus further demyelinating events in grey and white matter occur with subsequent axonal pathology. There is a high unmet medical need in MS patients to stop demyelination, enhance remyelination and prevent irreversible axonal pathology.

The cuprizone model is a toxin-induced demyelination model to study myelination processes in the CNS [22, 50, 57] without a major involvement of the peripheral immune system. The copper chelator cuprizone induces mitochondrial damage and the subsequent energy deficit in the highly energy-dependent ODs leads to apoptosis. OPCs are not affected by cuprizone intoxication and can readily proliferate and differentiate to produce new ODs with normal myelination efficiency. Thus, the cuprizone model is well suited to test regenerative treatment paradigms for MS that enhance remyelination independently of invading peripheral immune cells.

Microglia are the resident phagocytes of the brain that play an important role in modeling the CNS synaptic circuitry, keeping myelin homeostasis and removal of pathogens and cell debris ([17], for review see [38]). It has been shown that reduced neuroinflammation preserves axons [39] and that removal of myelin debris is essential to initiate repair by remyelination [4, 26, 40] in the cuprizone model.

The colony-stimulating factor 1 receptor (CSF1R) signaling pathway is essential for microglia survival [10, 11]. Several groups have shown that inhibition of the CSF1R pathway is neuroprotective in different disease conditions like amyotrophic lateral sclerosis [30], Charcot-Marie-Tooth type 1 neuropathies [23], catatonia in psychiatric disorders [19] and Alzheimer’s disease [29, 36, 48] by reducing neuroinflammation/inflammation. Here, we investigated the involvement of microglia in the de- and re-myelination processes in the murine cuprizone model. Mice were treated prophylactically and therapeutically with the CSF1R kinase inhibitor, BLZ945 [25, 41], to investigate the consequences of microglia depletion on de- and re-myelination events. Magnetic resonance imaging (MRI) was used to monitor in vivo the effects of cuprizone in several brain regions. Specifically, changes in magnetization transfer ratio (MTR) and signal intensity were used as markers of alteration in myelin levels induced by cuprizone, as confirmed by *post-mortem* histological analyses and in agreement with earlier work [12, 31, 49, 51, 56]. In both treatment paradigms BLZ945 enhanced myelination in certain brain regions, thus opening a new way for a novel therapy in treating myelination deficiencies in MS.

## Materials and methods

### Animals

Studies described in this report were approved by the Swiss Cantonal Veterinary Authority of Basel City, Switzerland, under the license numbers 2711 and 2119.

Mice (C57BL/6 J and C57BL/6 J OlaHsd) were commercially purchased from Charles River Laboratories (Sulzfeld, Germany), Harlan Laboratories BV (Horst, The Netherlands) or obtained from Novartis Pharma AG breeding colonies (8–9 weeks old, females). TREM2 knock-out mice were purchased from UCDavis KOMP Repository (Project ID VG10093) and were then bred at Novartis Pharma AG. Genotyping of TREM2 KO mice was performed according to the provided method. All the animals were allowed to adapt for 7 days prior to the start of the experiment and housed in IVC racks (max. 4 mice/XJ Type cage). The animals were given access to food and water ad libitum. Before killing animals were perfused trans-cardiac by phosphate-buffered saline (PBS) and then with 4% paraformaldehyde (PFA). The brains were subsequently isolated and fixed in 4% PFA for 48 h at 4 °C.

### Compound treatments

Animals were treated with cuprizone for 5 weeks. Cuprizone (Bis(cyclohexanone) oxaldihydrazone, Sigma-Aldrich, Buchs, Switzerland) was mixed into rodent food pellets (0.2% w/w) by Provimi Kliba AG (Kaiseraugst, Switzerland). Animals were treated with 169, 127, 100, 85, 60, 20 and 7 mg/kg of BLZ945, once per day (qd), per os (p.o.) 10 ml/kg. BLZ945 was prepared in 0.5% methylcellulose in water and 0.1% Tween-80.

### Magnetic resonance imaging (MRI)

Measurements were performed with a Biospec 70/30 spectrometer (Bruker Medical Systems, Ettlingen, Germany) operating at 7 T. The operational software of the scanner was Paravision 5.1 (Bruker). Images were acquired from anesthetized, spontaneously breathing animals using a mouse brain circularly polarized coil (Bruker, Model 1P T20063 V3; internal diameter 23 mm) for radiofrequency excitation and detection. Neither cardiac nor respiratory triggering was applied. Following a short period of introduction in a box, animals were maintained in anesthesia with 1.5% isoflurane (Abbott, Cham, Switzerland) in oxygen, administered via a nose cone. During MRI signal acquisitions, animals were placed in prone position in a cradle made of Plexiglas, the body temperature was kept at 37 ± 1 °C using a heating pad, and the respiration was monitored.

A T_2_-weighted, two-dimensional multislice RARE (Rapid Acquisition with Relaxation Enhancement) sequence [18] was used for determining the anatomical orientation and for evaluating signal intensities. This was followed by a two-dimensional multislice gradient-recalled FLASH (Fast Low-Angle Shot) acquisition [16] for assessment of MTR. As both sequences had the same anatomical parameters, the choice of the regions-of-interest for evaluations was performed on the RARE images and then transferred to the FLASH images. MRI images were analyzed using the ParaVision software.

The parameters of the acquisitions were the following: (a) RARE sequence: effective echo time 80 milliseconds (ms), repetition time 3280 ms, RARE factor 16, 12 averages, field of view 20 × 18 mm^2^, matrix size 213 × 192, pixel size 0.094 × 0.094 mm^2^, slice thickness 0.5 mm, 15 adjacent slices. Hermite pulses of duration/bandwidth 1 ms/5400 Hz and 0.64 ms/5344 Hz were used for radiofrequency excitation and refocusing, respectively. Fat suppression was achieved by a gauss512 pulse of 2.61 ms/1051 Hz duration/bandwidth followed by a 2-ms-long gradient spoiler. The total acquisition time was of 7 min 52.3 s; (b) FLASH sequence: echo time 2.8 ms, repetition time 252.8 ms, 4 averages, field of view 20 × 18 mm^2^, matrix size 213 × 192, pixel size 0.094 × 0.094 mm^2^, slice thickness 0.5 mm, 15 adjacent slices. A hermite pulse of 0.9 ms/6000 Hz duration/bandwidth and flipangle 30° was used for radiofrequency excitation. MTR contrast was introduced by a gauss pulse of 15 ms/182.7 Hz duration/bandwidth applied with radiofrequency peak amplitude of 7.5 μT and an irradiation offset of 2500 Hz. The acquisition was then repeated with the same parameters but without the introduction of the MTR contrast. MTR was then computed using the formula MTR = (S_0_-S_MTR_)/S_0_ where S_0_ and S_MTR_ represent respectively the signal intensities in the FLASH acquisitions without and with the introduction of the MTR contrast. The total acquisition time for both data sets was 6 min 31.6 s.

### Quantitative determination of BLZ945 in blood and brain

Sample preparation and analysis was based on a modified protein precipitation procedure followed by liquid chromatographic separation coupled with mass spectrometry for detection. Cerebella were homogenized in a gentleMACS™ Dissociator (Milteniy Biotec, # 130-093-235) by adding 20% CH_3_OH to a final concentration of 0.20 g/mL. Blood-EDTA samples were directly used for further preparation. Calibration, quality control, and recovery control samples were prepared by spiking blank blood and blank brain homogenate with known quantities of BLZ945 (between 0.02 and 62.5 μg/mL). For analyte determination, Labetalol hydrochloride (Sigma-Aldrich, #L1011) was used as generic internal standard (IS). Aliquots of 10 μL calibration standard, quality control, recovery control, and unknown samples were transferred to 0.75 ml 96- well- Loborack (Vitaris AG, # 51004 BC -MIC) and 3 μL IS mixture (2.5 μg/mL in 50% CH_3_CN) was added to each tube. For protein precipitation and extraction from the blood and brain matrix, 200 μl CH_3_CN was added. After vortexing for 10 min, the samples were centrifuged at 3220 *g* for 15 min at 4 °C. 50 μl of the upper layer was transferred to a 1.2 mL 96 deep well plate (Thermo Scientific, # AB-0787).

LC–MS–MS analysis: For quantitative analysis, a 1.5 μl aliquot of each sample, including calibration, quality control, and recovery control samples were injected with a cooled Waters™ Acquity Urbinary sample manager and HPLC system. The test article and its internal standard were separated with an ACE C18-PFP (50 × 2.1 mm ID, 3.0 μm pore size; # ACE-1110-0502) as column at 40 °C. For separation a linear gradient from 5 to 65% B in 1.7 min at a flow rate of 0.400 mL/min was applied. The total cycle time was 3.5 min. The mobile phase used was A: water with 0.1% formic acid, and B: CH_3_CN with 0.1% formic. For detection the column effluent was directly guided in a AB Sciex API5500 Triple quad mass spectrometer equipped with a TurboIonSpray™ interface. The detection was done in MRM positive ion mode. Quantification was based on the compound/IS ratio of the extracted ion chromatograms of the selected mass transitions 399 m/z → 242 m/z for BLZ945 and 329 m/z → 162 m/z for Labetalol (IS). The unknown sample concentration was calculated using external calibration curves. The LLOQ of the method was set to 20 ng/mL for blood, and 100 ng/mL for brain samples, and the recovery from the matrix was 95 ± 2%. All calculations were performed with AB Sciex Analyst software 1.6.2.

### Histology of brains

After fixation brains were processed for paraffin embedding by dehydration through increasing ethanol series. Automated immunohistochemistry of paraffin sections was performed on 3 μm paraffin sections mounted on SuperFrost+ slides (Thermo Fisher Scientific) and automatically immunostained using the Discovery XT technology (Ventana, Roche Diagnostics). Sections were deparaffinized, rehydrated, subjected to antigen retrieval by heating with CC1 cell conditioning buffer for 28–68 min according to the antibody, incubated for 1–3 h according to the antibody at room temperature with primary antibody diluted in antibody diluent (Ventana), incubated with the respective biotinylated secondary antibody diluted in antibody diluent, reacted with DABMab kit and counterstained with Hematoxylin II and Bluing reagent (Ventana). Slides were washed with soap in hot tap water and rinsed under cold running tap water to remove the soap, then dehydrated and embedded with Pertex.

For LFB staining, slides were deparaffinized and rehydrated to 95% ethanol. Slides were then incubated in LFB solution (Solvent Blue 38 (Sigma S3382) in 95% ethanol and 10% acetic acid (Sigma 695092)) overnight at 60 °C, rinsed in 95% ethanol for 1 min, then in distilled water for 2 min and in 0.05% lithium carbonate for 5 s. Subsequently, slides were rinsed in 70% ethanol twice for 10 s, then in distilled water for 2 min. The rinsing was repeated in 0.05% lithium carbonate (Merck 105680) prepared freshly, 70% ethanol and distilled water until there was a sharp contrast between the blue of the white matter (myelin) and the colorless grey-matter. Finally, slides were dehydrated starting with 95% ethanol and mounted in Pertex.

For Ki67 and Iba1 immunofluorescence co-staining, 5 μm-thick sections were de-paraffinized, rehydrated and subjected to antigen retrieval in 10 mM citrate buffer pH 6. After blocking with 10% normal donkey serum (NDS) in 1xTBS for 20 min, sections were incubated with monoclonal IgG rabbit anti-mouse ki67 antibody overnight at 4 °C. Sections were then washed twice in TBS and incubated for 30 min at RT with biotin donkey anti-rabbit IgG (H + L). After washing twice in TBS, slides were incubated with Streptavidin, Alexa Fluor™ 546 conjugate for 30 min at RT, washed again twice in TBS and blocked in 10% NDS for 20 min at RT. This was followed by incubation with goat anti-Iba1 antibody for 1 h at RT, washing steps in TBS and incubation with donkey anti-goat IgG Alexa Fluor™ 488 for 30 min at RT. After washing in TBS, nuclei were stained with DAPI (Thermo Fisher Scientific R37606) for 10 min at RT, washed and slides were mounted with MOWIOL 4–88 Reagent solution (Merck, 475904).

### Histology on the spinal cord

Lumbar spinal cord (L1-L3 spinal segments) samples were carefully dissected with the corresponding vertebrae and fixed in 10% neutral buffered formalin (Sigma-Aldrich, HT501128) for 48 h. Tissues were then transferred to decalcifying reagent Immunocal® (StatLab, 1414-32; StatLab Medical Products) for 96 h. Dehydration and paraffin infiltration were performed on a Tissue-Tek VIP 6 Vacuum Infiltration Processor (Sakura Finetek Europe). Following paraffin embedding, 5 μm-thick sections of spinal cord samples were cut and stained with Iba1 antibody, as described for brain sections.

### Antibodies

Primary antibodies are: rabbit anti-mouse MBP (Dako A0623) 1:1000; rabbit anti-mouse GST-π (MBL 312) 1:500; Rabbit anti-Iba1 (Wako 019–19741, 50 μg/100 μl) 1:500; rabbit anti-GFAP (Dako Z0334) 1:5000; rabbit anti-mouse MOG (abcam ab32760) 1:100; rabbit anti-dMBP (Millipore AB5864) 1:3000; mouse anti-Neurofilament (Covance SMI312) 1:5000; rabbit anti-mouse ALDH1L1 (abcam ab87117) 1:1000; rabbit anti-mouse NeuN (Millipore ABN78) 1:2000; rat anti-mouse CD107a (Biorad MCA4707T) 1:200; rabbit anti-mouse NG2 (abcam ab129051) 1:200; rabbit anti-mouse ki67 (abcam ab16667, clone SP6) 1:100; goat anti-Iba1 (Thermo Fisher Scientific PA5–18039) 1:250.

Secondary detection antibodies are: Goat anti-rabbit IgG biotinylated (Jackson ImmunoResearch 111–065-144) 1:1000; Goat anti-rabbit IgG biotinylated (Vector BA-1000) 1:200 or 1:1000; Goat anti-mouse IgG biotinylated (Vector BA-9200) 1:1000; Goat anti-rat biotinylated (Vector BA-9400) 1:200; donkey anti-rabbit IgG (Jackson ImmunoResearch 711–065-152) 1:200; Streptavidin, Alexa Fluor™ 546 conjugate (Thermo Fisher Scientific S11225) 1:800; donkey anti-goat IgG Alexa Fluor™ 488 (Thermo Fisher Scientific A11055) 1:500.

### Analysis of histological images

For the quantitative evaluation of microglia/astrocyte numbers and morphology based on image analysis from histological stained brain sections, a proprietary image analysis platform (ASTORIA, Automated Stored Image Analysis, Novartis Pharma AG) was developed based on MS Visual Studio 2010 and many functions from Matrox MIL V9 libraries (Matrox Inc).

For the detection and analysis of soma, proximal and distal processes, the following sequence of steps was performed (Additional file 1: Figure S14): 1. Slides with brain sections for assessment of (brown) immunohistochemically stained microglia (Iba1) or astrocyte (GFAP) soma and their proximal and distal processes were scanned with Aperio’s Scanscope (Leica Biosystems AG) at 20× magnification (Additional file 1: Figure S14A). 2. Each image was processed using the ImageScope software (V12.1.0.5029, Aperio, Leica Biosystems AG) according to the following steps: A: color deconvolution to obtain brown staining without blue; B: segmentation of brain tissue from white background through thresholding, morphological closing, filling of holes, opening and elimination of too small objects, resulting in a binary mask of the valid tissue and sample area; C: adaptive thresholding for the individual segmentation of soma, based on the average gray value of the blue channel of the color-deconvoluted brown image at sufficiently dark regions (indicative for soma). The computed threshold was used for binarization, and after size filtering yielded the soma mask image (within the valid sample region, Additional file 1: Figure S14B); D: segmentation of processes through morphological tophat transformation with a size to pick thin processes. Adaptive thresholding was applied again to segment the processes (using the previously determined gray average of brown objects), followed by binarization of the top hat image and size filtering of the resulting objects; E: subtraction of soma (that may also have been picked by top hat thresholding) to obtain an image mask of true processes (Additional file 1: Figure S14C); F: ultimate thinning of processes for length computation; G: proximal processes: A predefined number of dilations of soma was used to define a reference (marker) region for proximal soma, employing a circle around the soma center to define the cutoff boundary for proximal processes. Thinned proximal processes with marker in dilated soma and limited by circular influence zone (set of “proximal thinned processes”) were then reconstructed around the soma center. “Final proximal processes” were collected through reconstruction of all processes having markers in the “proximal thinned processes” set (Additional file 1: Figure S14D); H: soma was added to proximal processes to obtain a set of “visible microglia”; I: Distal processes: Reconstruction of processes from proximal processes only (i.e. ignoring those in background or from soma in different focus plane), then subtract circular region defining proximal processes, to yield set of distal processes (Additional file 1: Figure S14E); J: in the optical density computation for soma as well as “visible microglia” (individual soma+proximal processes complex within circular reference region, Additional file 1: Figure S14F), local background (non-visible microglia) was used for reference; K: morphometric features (size, form factor, length) were computed for soma, proximal and distal processes (Additional file 1: Figure S14G).

These image analysis algorithms were also used to quantify SMI312, dMBP, GST-π, MBP and NeuN stained sections according to the above description.

For MBP an alternative quantification with ImageJ analyzing IntDen (integrated density) with threshold was performed in addition to the one described above.

### MOG peptide-induced experimental autoimmune encephalomyelitis (EAE)

Female C57BL/6 mice (Harlan, Itingen, Switzerland; n = 30) were immunized with a subcutaneous injection of rat myelin oligodendrocyte glycoprotein peptide (MOG1–125; in house produced; 200 μg/100 μl) emulsified in 4 mg/ml complete Freund adjuvant (CFA, Sigma-Aldrich) in the lower back. Pertussis toxin (200 ng per mouse; Sigma-Aldrich) was administered intraperitoneally on days 0 and 2.

Evaluation of EAE: The mice were observed and weighed daily. They were assessed for clinical signs on a scale from 0 to 5, with graduations of 0.5 for intermediate scores. 0: no clinical signs, 1: Limp tail/complete loss of tail tonus; 2: Clear hind limb weakness; 3: Complete bilateral hind limb paralysis; 4: Fore limbs and hind limbs paralysis; 5: Death. Supplementary food and gel food were provided inside the cage once first clinical signs appeared.

### Gene expression

Lysing Matrix D tubes (MP Biomedicals, 116913500) were used for lysis of lumbar spinal cord (L4-L6 spinal segment) in 1 ml of TRIzol reagent (Thermo Fisher Scientific, 15596018). Total RNA was then isolated with TRIzol reagent according to the manufacturer’s instructions and a TURBO DNA-free™ Kit (Thermo Fisher Scientific, AM1907) was used for complete digestion of DNA.

RNA (500 ng) was reverse transcribed to cDNA using the High-Capacity RNA-to-cDNA™ Kit (Thermo Fisher Scientific, 4387406).

Quantitative PCR (qPCR) was carried out using 10 ng of cDNA per each sample and TaqMan® Universal PCR Master Mix (Thermo Fisher Scientific, 4324018). Gene-specific primers with FAM-labeled probes were from Thermo Fisher Scientific: Aif1, Mm00479862_g1; Csf1r, Mm01266652_m1; Cx3cr1, Mm02620111_s1; Tmem119, Mm00525305_m1; Trem2, Mm04209424_g1. Gapdh-specific primers (Mm99999915_g1) were used as internal control.

PCR cycling conditions on software supplied with ViiA™ 7 System (Applied Biosystems) were as follows: 50 °C for 2 min, 95 °C for 10 min, 40 cycles at 95 °C for 15 s, and 60 °C for 1 min. Data were expressed as Ct values and used for the relative quantification of targets with the ΔΔCt calculation to give N-fold differences. Data were transformed through the eq. 2^-ΔΔCt^.

## Results

### The CSF1R kinase inhibitor BLZ945 therapeutically enhances remyelination in the cuprizone model

**Fig. 1:**
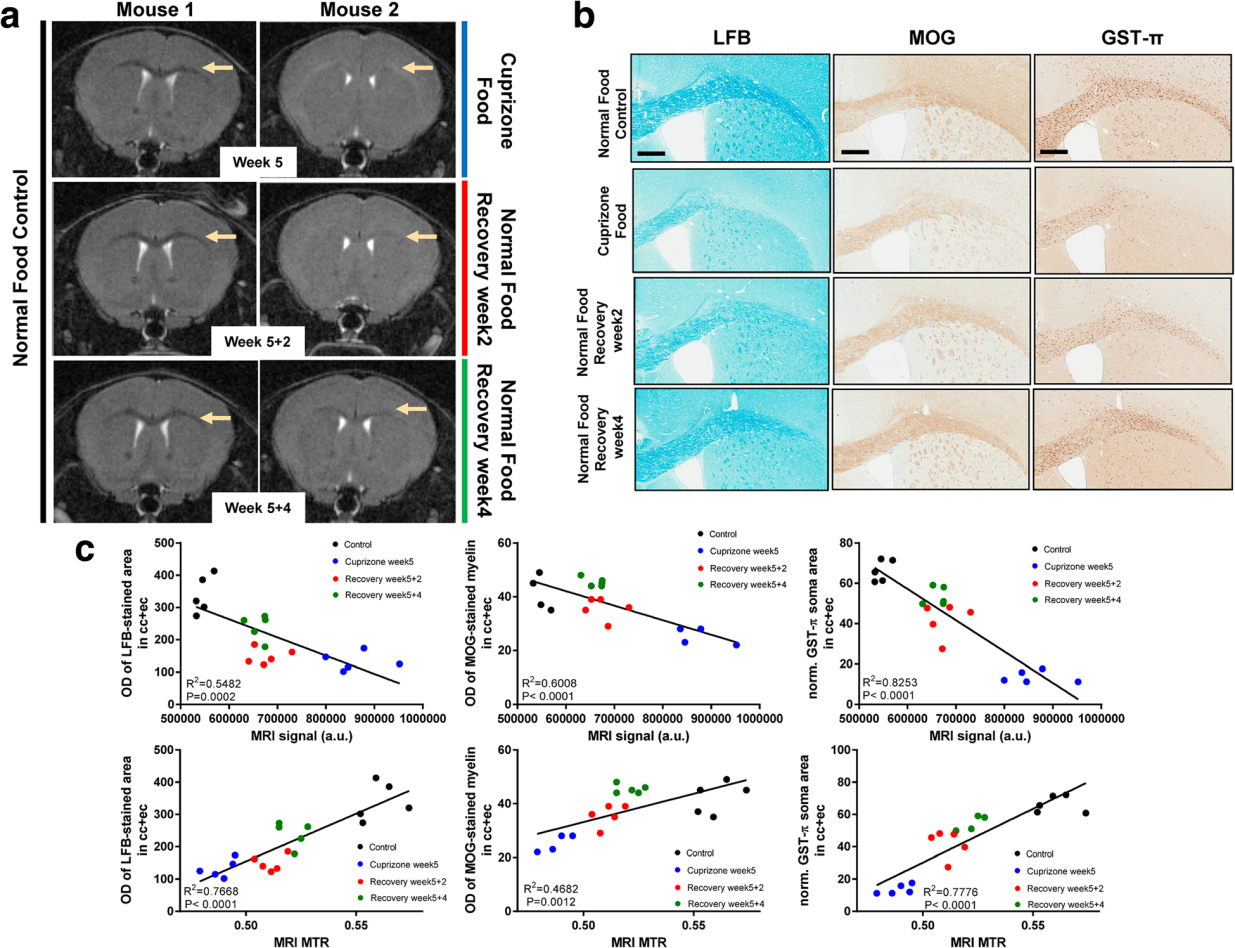
MRI reliably detects de- and re-myelination events in the cuprizone model in the corpus callosum/external capsule, correlating with myelin and oligodendrocyte histology. **a** Representative MRI images acquired from two mice, one receiving normal food (left) and the other treated with 0.2% cuprizone for 5 weeks with subsequent switch to normal food. **b** Representative pictures from histological and immunohistological stainings from the corpus callosum/external capsule after 5-week 0.2% cuprizone food and control food treatment and recovery for 2 and 4 weeks (switch to normal food) of C57BL/6 mice detecting myelin by Luxol fast blue (LFB), myelin oligondendrocyte glycoprotein (MOG) and GST-π positive oligodendrocyte cells in the corpus callosum/external capsule. **c** Correlation analysis of quantitative histology (LFB and MOG optical density (OD), GST-π positive soma area normalized (norm.) to region of interest) and MRI signal/MTR parameters. Two-tailed Pearson correlation analysis, correlation coefficients (R^2^) and *p* values are indicated. Scale bars: 400 μm. cc: corpus callosum, ec: external capsule, MRI: magnetic resonance imaging, MTR: magnetization transfer ratio, a.u.: arbitrary units, OD: optical density

The cuprizone model is ideal to analyze myelination processes in the CNS. We used 0.2% cuprizone in mice feed for 5 weeks to induce a strong demyelination together with a massive involvement of microglia and astrocytes as observed by histology (Fig. 1 and Additional file 1: Figure S1). We implemented a longitudinal, non-invasive magnetic resonance imaging (MRI) method to measure myelination in the brain (Fig. 1a) and correlated the MRI parameters with quantitative histological readouts in brain (Fig. 1b, c and Additional file 1: Figure S2) similar to what has been described by others [5, 49]. After stop of feeding cuprizone and change to normal food for 2 and 4 weeks recovery spontaneous remyelination by MRI could be observed (Fig. 1a and Additional file 1: Figure S2a). Quantitative histological readouts for myelin (Luxol Fast Blue (LFB), myelin oligodendrocyte glycoprotein (MOG)) and oligodendrocytes (ODs, GST-π) revealed robust reduction after 5-week intoxication and reappearance during the 2 and 4 weeks recovery phase (Fig. 1b and Additional file 1: Figure S2b). All histological quantifications correlated significantly with the MRI parameters at baseline, at maximal cuprizone-induced pathology and during recovery (Fig. 1c).

We next tested the CSF1R kinase inhibitor, BLZ945, in this model at a high dose of 169 mg/kg p.o. This dose has been chosen based on studies in naïve mice, revealing that BLZ945 administered daily p.o. during 5 days dose-dependently deplete microglia in the brain cortex and spinal cord (Additional file 1: Figure S3 and S4). After 4–5 days microglia depletion was maximal at the high dose of 169 mg/kg (Additional file 1: Figure S3b). After treatment stop microglia re-appeared readily already after 3 days and reached normal numbers at 7 days (Additional file 1: Figure S3b). Microglia activation (microglia soma and proximal processes normalized to distal processes) was analyzed by morphological characteristics via image analysis of Iba1-immunostained brain sections (see Material and Methods and Additional file 1: Figure S14). BLZ945 treatment induced a slight increase in activation status of the remaining microglia. However, newly generated microglia after 3 days BLZ945 removal were highly activated, while they reached normal levels after 7 days (Additional file 1: Figure S3b). Furthermore, microglia activation was dose-dependent but bell-shaped that was highest at a medium dose of 60 mg/kg BLZ945 (Additional file 1: Figure S3c). Microglia depletion in the spinal cord was additionally confirmed on gene expression level (Additional file 1: Figure S4b).

**Fig. 2:**
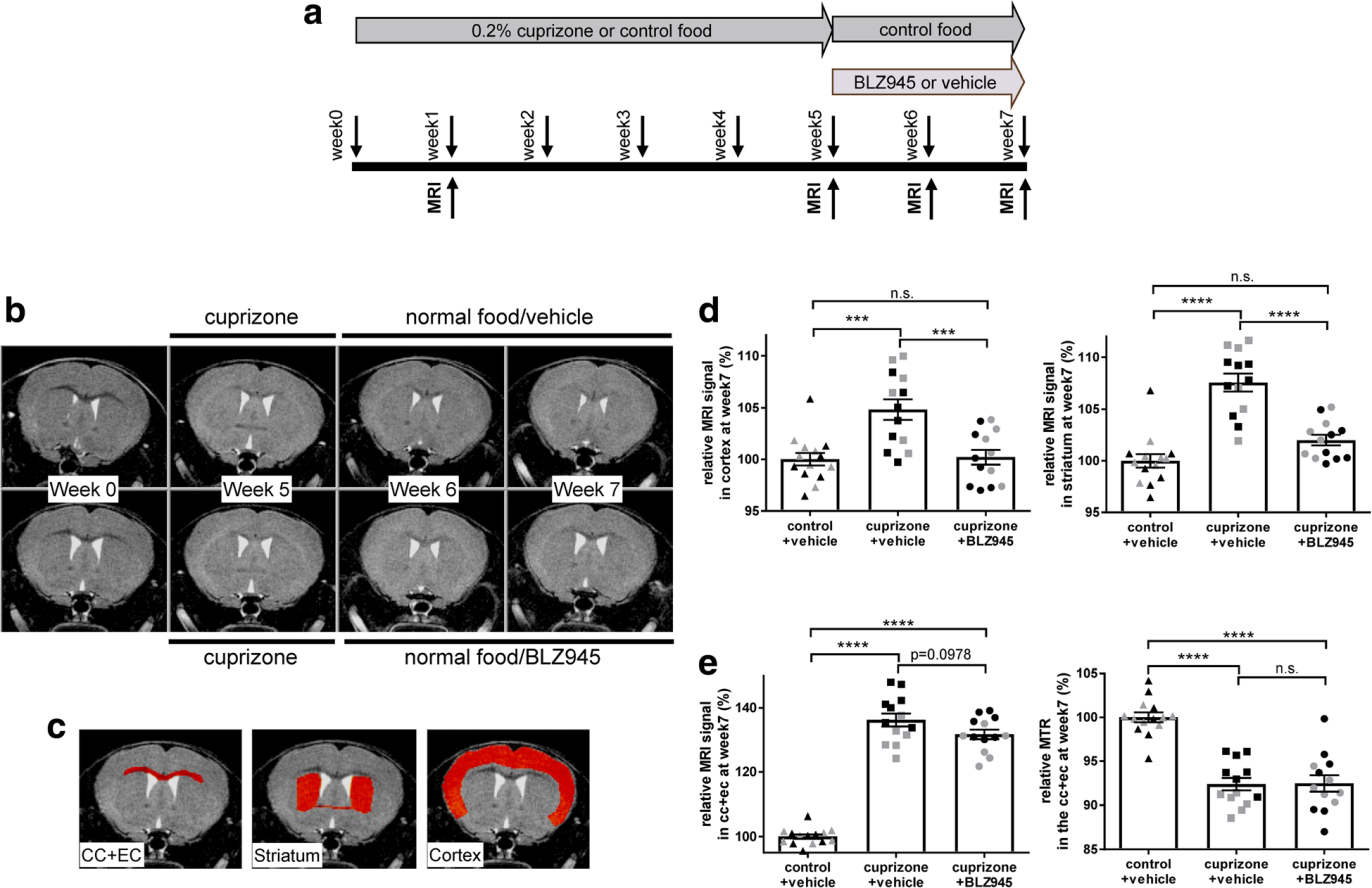
A 2-week therapeutic treatment with BLZ945 after a 5-week cuprizone intoxication period reduced MRI signal in cortex and striatum but not corpus callosum, suggesting increased remyelination. **a** Schematic diagram of the experimental setup for the therapeutic treatment. Groups consisted of mice treated for 5 weeks with control food (normal food) or 0.2% cuprizone in food and then switched back to control food (normal food) for the 2-week therapeutic treatment (vehicle or BLZ945 169 mg/kg p.o., qd). MRI measurements were performed at week 0 (baseline), week 5 at max. Pathology of cuprizone intoxication, at week 6 (1 week of therapeutic or vehicle treatment on control food) and at week 7 (2 weeks of therapeutic or vehicle treatment on control food). Mice were killed at week 7 immediately after the last MRI measurement. **b** Representative MRI images acquired from two mice, one receiving 0.2% cuprizone and then normal food with vehicle (upper row) and the other treated with 0.2% cuprizone for 5 weeks with subsequent switch to normal food and BLZ945 treatment (lower row). **c** Representative MRI images indicating analyzed brain regions (in red). **d** MRI signal in cortex and striatum for the different treatment groups. For each brain region, MRI signal was normalized to absolute values in the control group (control food, vehicle treatment). **e** MRI signal and MTR in corpus callosum and external capsule for the different treatment groups (normalized to values in the control group). Because of the small magnitude (≤2%) of MTR reductions in the cortex and striatum following the 5-week cuprizone intoxication period, MTR changes in these areas were not considered here. Grey and black symbols indicate individual values from two independent experiments. Group sizes: control+vehicle (*n* = 7 from experiment 1, *n* = 7 from experiment 2), cuprizone+vehicle (*n* = 6 from experiment 1, *n* = 6 from experiment 2; one mouse was removed from experiment 2 due to technical reasons), cuprizone+BLZ945 (n = 7 from experiment 1, *n* = 6 from experiment 2). Data is shown as mean ± SEM. Statistics (for combined experiments): Turkey’s multiple comparison test (***: *p* < 0.001, ****: *p* < 0.0001), n.s.: not significant, ctrl: control, cpz: cuprizone, cc: corpus callosum, ec: external capsule, MRI: magenetic resonance imaging, MTR: magnetization transfer ratio

BLZ945 treatment for 2 weeks with normal food after induction of demyelination for 5 weeks with 0.2% cuprizone (Fig. 2a) showed a significant effect in the cortex and striatum (relative to that in control mice, see Fig. 2c for the region-of-interests used for MRI quantification) as measured by in the MRI in two independent experiments (Fig. 2b). For both brain areas, the MRI signal in BLZ945-treated animals almost normalized to levels of control mice, whereas the MRI signal of cuprizone-fed, vehicle-treated mice was still enhanced as compared to that in control mice. This effect was highly significant only after 2 weeks of BLZ945 treatment (Additional file 1: Figure S5a, b). No effect of BLZ945 was observed in the corpus callosum and external capsule in two independent experiments at any time-point (Fig. 2e and Additional file 1: Figure S5c, d), as evidenced by both the MRI signal intensity and MTR. In this therapeutic experiment, mice were randomized according to the responses detected by MRI at week 5 of maximal cuprizone intoxication, just before beginning of vehicle or BLZ945 treatment, to obtain homogenous groups at the start of the treatment.

**Fig. 3:**
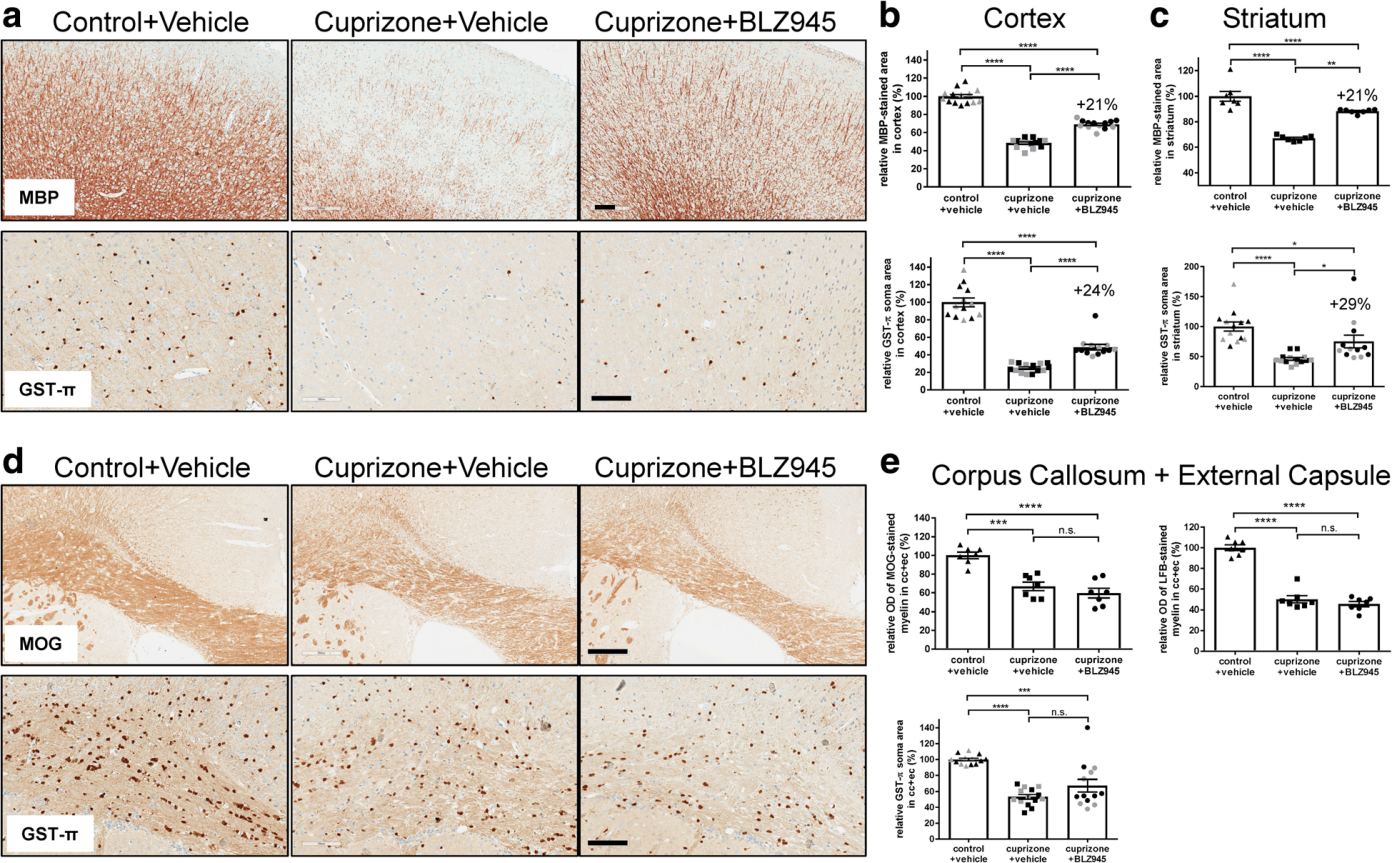
A 2-week therapeutic treatment with BLZ945 after a 5-week cuprizone intoxication period enhanced remyelination and increased the number of mature oligodendrocytes in cortex and striatum but not corpus callosum/external capsule compared to vehicle treatment. **a** Representative pictures from immunohistological stainings detecting myelin basic protein (MBP) and mature oligodendrocytes positive for GST-π in the cortex for the different treatment groups at week 7 (see Fig. 2a for the experimental setups and groups). **b, c** Corresponding quantitative analysis of the immunohistochemistry for MBP (stained area) and GST-π (number of positive cells) in the cortex and striatum normalized to values from control vehicle mice. **d** Representative pictures from immunohistological stainings detecting myelin oligondendrocyte glycoprotein (MOG) and mature oligodendrocytes positive for GST-π in the corpus callosum and external capsule for the different treatment groups at week 7.** e** Corresponding analysis of the immunohistochemistry for MOG (optical density, OD) and GST-π (number of positive cells) as well as OD analysis of Luxol fast blue (LFB) in the cc and ec. Values were normalized to those of control vehicle mice. Grey and black symbols indicate individual values from two independent experiments. Group sizes: control+vehicle (n = 7 from experiment 1, n = 6–7 from experiment 2), cuprizone+vehicle (*n* = 7 from experiment 1, n = 7 from experiment 2), cuprizone+BLZ945 (n = 7 from experiment 1, *n* = 5–6 from experiment 2). Data are shown as means±SEM. Scale bars: 200 μm (MBP and MOG), 100 μm (GST-π). Statistics (for combined experiments): Turkey’s multiple comparison test one-way ANOVA (*: *p* < 0.05, **: *p* < 0.01, ***: *p* < 0.001, ****: *p* < 0.0001, n.s.: not significant), cpz: cuprizone, cc: corpus callosum, ec: external capsule, OD: optical density

The positive effect in the MRI parameters observed after 2 weeks of therapeutic BLZ945 treatment could be confirmed in immunohistochemistry of paraffin-embedded brain sections and quantitative image analysis (Fig. 3). Myelin basic protein (MBP) and ODs (GST-π) were significantly increased after BLZ945 treatment compared to vehicle control in the cortex (Fig. 3a, b and Additional file 1: Figure S6a-b) and striatum (Fig. 3c and Additional file 1: Figure S6a). However, the extent of MBP-positive remyelination and GST-π-positive OD numbers did not reach control levels after 2 weeks of BLZ945 treatment. Furthermore, the absence of any enhancement of remyelination in the corpus callosum and external capsule as observed by MRI could be verified by histology (Fig. 3d, e). There was a marked reduction of MOG, ODs (GST- π) as well as myelin based on Luxol Fast Blue (LFB) staining after cuprizone treatment in the vehicle group compared to controls but no difference following 2 weeks of therapeutic BLZ945 treatment (Fig. 3d, e). Important to note that BLZ945 treatment substantially reduced the NG2-positive oligodendrocyte precursor cells (OPCs) (Additional file 1: Figure S6c) similar as reported elsewhere [17]. This indicates that either the NG2-positive OPCs differentiated readily to mature ODs upon BLZ945 treatment and/or that NG2-negative precursor cells exist as a pool for ODs.

**Fig. 4:**
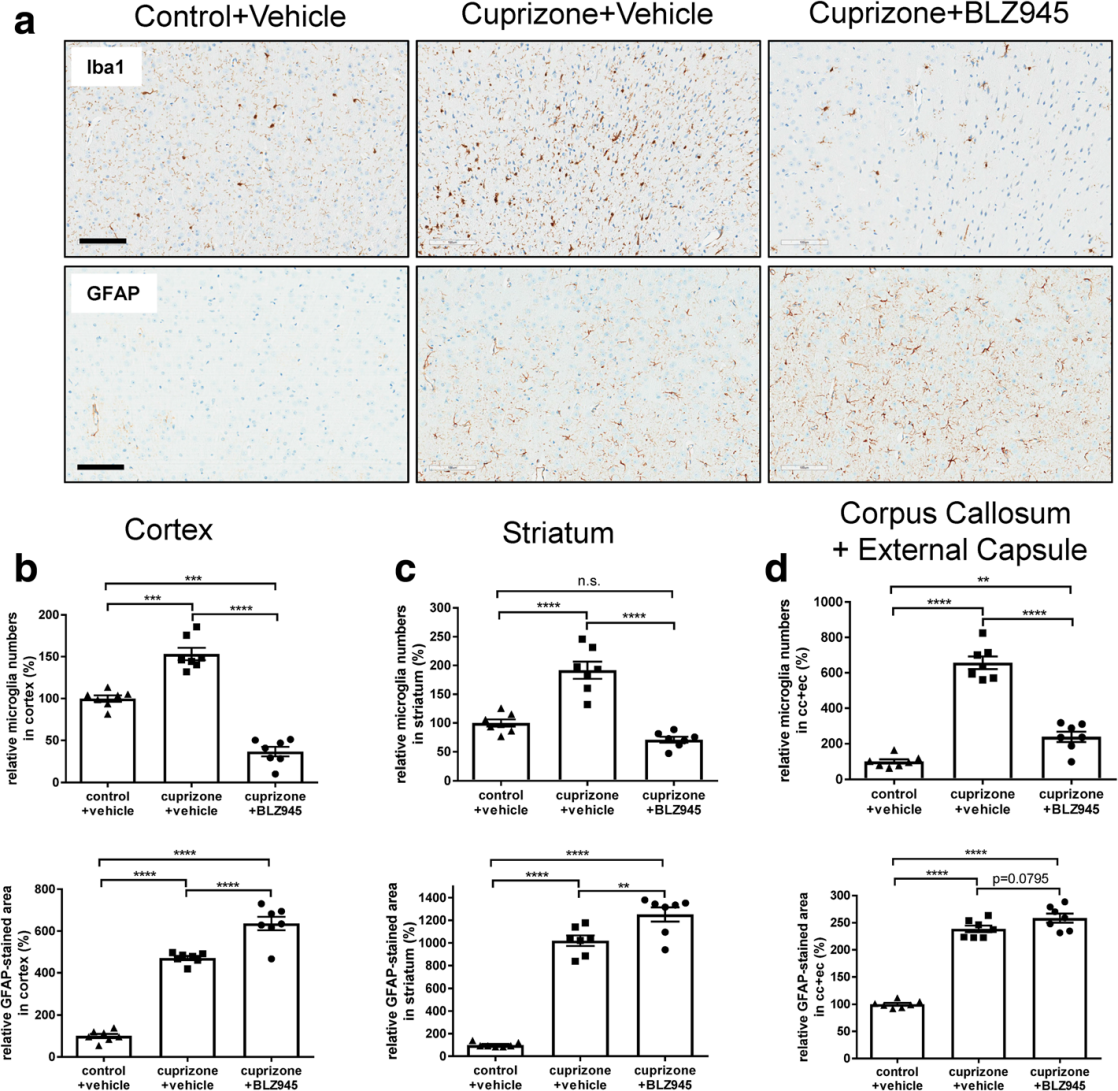
A 2-week therapeutic treatment with BLZ945 after a 5-week cuprizone intoxication period reduced microglia numbers but enhanced astrocytes. **a** Representative pictures from immunohistological stainings detecting the microglia marker Iba1 and glial fibrillary acidic protein (GFAP) astrocytes in the cortex for the different treatment groups at week 7 (see Fig. 2a for the experimental setup and groups). **b, c, d** Corresponding quantitative analysis of the immunohistochemistry for Iba1-positive microglia numbers and GFAP-positive astrocyte stained area in the cortex, striatum and corpus callosum/external capsule. Values were normalized to those of control, vehicle-treated mice. Group sizes: For all treatments *n* = 7. Data are shown as means±SEM. Scale bars: 100 μm. Statistics: Turkey’s multiple comparison test one-way ANOVA (**: *p* < 0.01, ***: *p* < 0.001, ****: *p* < 0.0001, n.s.: not significant), cpz: cuprizone, cc: corpus callosum, ec: external capsule

Microglia and astrocytes were highly activated and increased after 5-week cuprizone-induced demyelination in all brain areas examined (Fig. 4 and Additional file 1: Figure S7). In the cortex and striatum the numbers of Iba1-positive microglia were 1.5–2-fold higher than in control mice (Fig. 4b, c), whereas in the corpus callosum and external capsule this increase was 6-fold (Fig. 4d). BLZ945 significantly reduced the number of Iba1-positive microglia compared to cuprizone-challenged, vehicle-treated animals in all brain areas analyzed (Fig. 4). Moreover, compared to control+vehicle treatment, BLZ945-treated mice after cuprizone intoxication showed a lower number of Iba1-positive microglia in the cortex and a trend towards lower number in the striatum (Fig. 4b, c) but not in the corpus callosum and external capsule (Fig. 4d). However, this reduction in Iba1-positive microglia with BLZ945 in the cortex of cuprizone mice was much lower than that observed in naïve animals (Additional file 1: Figure S3). In cortex and striatum the GFAP-positive astrocytes were increased 5–6-fold compared to control (Fig. 4b, c) whereas in the corpus callosum and external capsule this increase was only 2–2.5-fold higher (Fig. 4d). Similar results for enhanced astrocytosis could be observed with the alternative astrocyte marker ALD1L1 (Additional file 1: Figure S8). We did not observe any astrocyte proliferation by GFAP and Ki67 co-immunofluorescence staining in any brain area (data not shown). Interestingly, the extent of increase of microglia and astrocytes in cortex, striatum and corpus callosum/external capsule seemed to occur reciprocally. In cortex and striatum the increase of microglia (1.5–2-fold) was not as extensive as the increase of astrocytes (4–6-fold) whereas in the corpus callosum/external capsule it was the opposite, the increase of Iba1-positive microglia was much higher (6-fold) than that of astrocytes (2.5-fold). However, BLZ945 treatment for 2 weeks after cuprizone feeding even further increased astrocyte numbers in all brain areas compared to vehicle (Fig. 4). This astrocyte increase was substantially higher in cortex and striatum than that in corpus callosum/external capsule. Morphological microglia image analysis to determine size and activation status (Additional file 1: Figure S14) revealed brain region-specific differences after therapeutic BLZ945 treatment (Additional file 1: Figure S7). Microglia size (microglia soma and proximal processes area normalized to microglia soma numbers, Additional file 1: Figure S7a), microglia form factor (Additional file 1: Figure S7b) and microglia activation (microglia soma and proximal processes area normalized to microglia distal processes area, Additional file 1: Figure S7c) were mostly increased after cuprizone treatment in all brain areas analyzed. Therapeutic BLZ945 treatment even further enhanced these microglia parameters in cortex and striatum revealing a higher morphological activation status of the remaining microglia. The meaning of this is for now uncertain, but it could either relate to higher microglia functionality like phagocytosis or ongoing microglia turnover. In corpus callosum and external capsule, however, these parameters were not changed at all or even reduced.

To investigate the relevance of microglia in an autoimmune disease model we therapeutically treated EAE mice, a model of human MS. Animals received two different doses (85 and 127 mg/kg, p.o., qd) of BLZ945 starting at near maximal disease 14 days after immunization (Additional file 1: Figure S9a). Treatment lasted until the end at day 28 post-immunization. No difference in clinical score for both BLZ945 groups compared to vehicle could be observed (Additional file 1: Figure S9a). The onset of EAE clinical pathology around 11–12 days post-immunization as well as the weight change was similar between the treatment and vehicle groups. Microglia depletion could be observed in the spinal cord gray matter at day 28 with treatment of 127 mg/kg BLZ945 (Additional file 1: Figure S9b, c). However, no microglia reduction after high dose BLZ945 treatment could be detected in the cortex (Additional file 1: Figure S9b, d). The microglia displayed a higher activation status in spinal cord and cortex (Additional file 1: Figure S9b, c, d). Furthermore, microglia in the cortex were highly proliferating as shown by Iba1 and Ki67 co-immunofluorescence staining (Additional file 1: Figure S9e); this was not observed in the cortex of vehicle-treated EAE mice (data not shown). This contradicts the mechanism of action of BLZ945, but as the microglia are confronted with peripheral immune cells in the EAE model their phenotypic status could be altered and other additional factors might contribute in regulating microglia homeostasis independent of the CSF1R pathway. Further work is needed to understand the microglia phenotype in the EAE model. Nevertheless, CSF1R kinase inhibition does not alter disease progression in the EAE model, a peripherally driven neuroinflammation model.

In summary, therapeutic BLZ945 treatment enhanced remyelination in specific brain regions after 5-week cuprizone intoxication, like cortex and striatum but did not change remyelination processes in the corpus callosum/external capsule.

### The CSF1R kinase inhibitor BLZ945 prophylactically prevents demyelination in the cuprizone model selectively in the corpus callosum but enhances myelin debris and axonal pathology in the external capsule and cortex

**Fig. 5:**
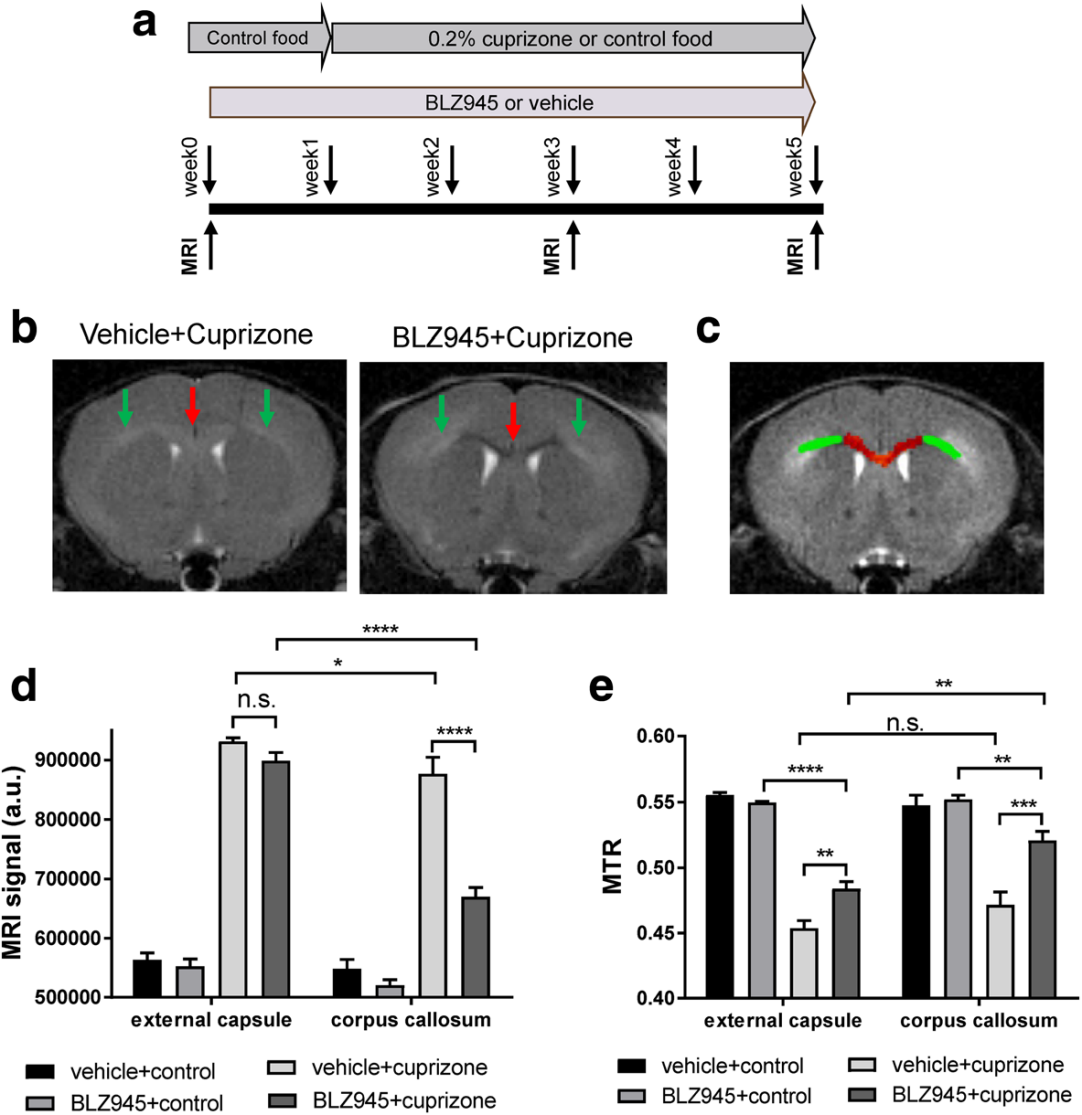
Prophylactic treatment with BLZ945 1 week before and during 5-week cuprizone intoxication inhibited demyelination in the corpus callosum but not in the external capsule. **a** Schematic diagram of the experimental setup for the prophylactic treatment. Groups consisted of mice pretreated for 1 week with vehicle or 169 mg/kg BLZ945 (p.o., qd) on normal food. Control food was continued or then switched to 0.2% cuprizone with concomitant continuation of vehicle or 169 mg/kg BLZ945 (p.o., qd) treatment for 5 weeks. MRI measurements were performed at week 0 (baseline) as well as week 3 and week 5 during cuprizone intoxification. Mice were killed at week 5 immediately after the last MRI measurement. **b** Representative MRI brain images for mice treated with either BLZ945 (p.o., qd, 169 mg/kg) or vehicle before (1 week) and during 0.2% cuprizone intoxification for 5 weeks. red arrows: corpus callosum, green arrows: external capsule. **c** Representative MRI brain images indicating the analyzed brain regions (red: corpus callosum, green: external capsule). **d, e** Quantification of the MRI contrast and MTR in the corpus callosum for the different treatment groups showing reduced MRI contrast and enhanced MTR for the corpus callosum compared to the external capsule. For all groups: *n* = 5. Data is shown as mean ± SEM. Statistics: Turkey’s multiple comparison test one-way ANOVA (*: *p* < 0.05, **: *p* < 0.01, ***: *p* < 0.001, ****: *p* < 0.0001), n.s.: not significant, a.u.: arbitrary units, MRI: magnetic resonance imaging, MTR: magnetization transfer ratio

**Fig. 6:**
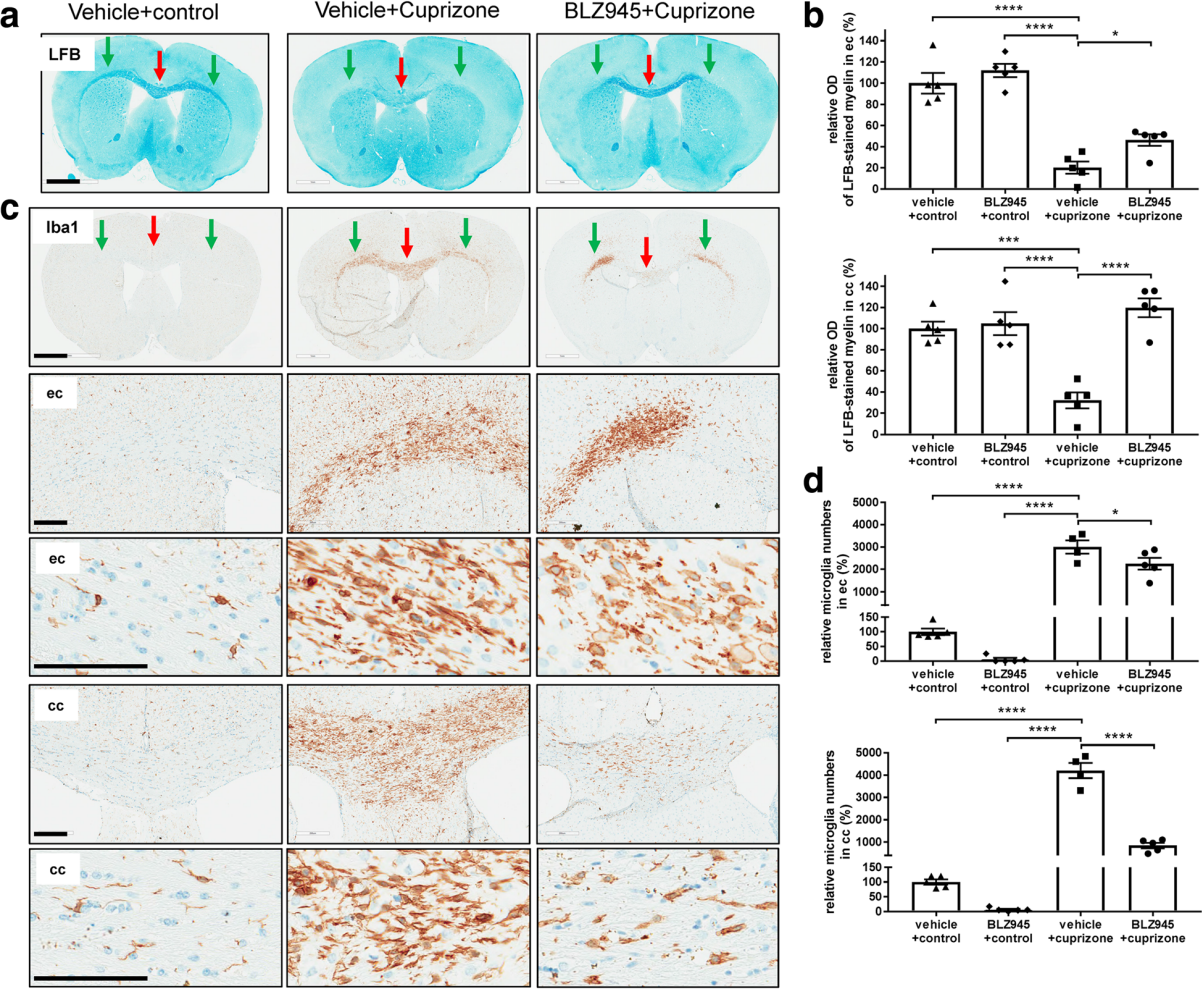
Prophylactic treatment with BLZ945 1 week before and during 5-week cuprizone intoxication inhibited demyelination and reduced microglia in the corpus callosum but enhanced axonal pathology and myelin debris in the external capsule. **a** Representative overview pictures from histological stainings of Luxol Fast Blue (LFB) for the different treatment groups at week5 (see Fig. 5a for the experimental setup and groups), red arrows: corpus callosum, green arrows: external capsule. **b** Corresponding analysis of the optical density (OD) of Luxol fast blue (LFB) in the cc and ec. **c** Representative overview and higher magnification pictures from immunohistological stainings detecting Iba1-positive microglia for the different treatment groups at week5 (see Fig. 5a for the experimental setup and groups), red arrows: corpus callosum, green arrows: external capsule. **d** Corresponding quantitative analysis of the immunohistochemistry for Iba1-positive microglia numbers in the corpus callosum and external capsule. Values were normalized to those of control vehicle mice. Group sizes: for all treatment groups *n* = 4–5. Data are shown as means±SEM. Scale bars: 200 μm for the higher magnification. Statistics: Turkey’s multiple comparison test one-way ANOVA (*: *p* < 0.05, ***: *p* < 0.001, ****: *p* < 0.0001), cpz: cuprizone, cc: corpus callosum, ec: external capsule, OD: optical density

**Fig. 7:**
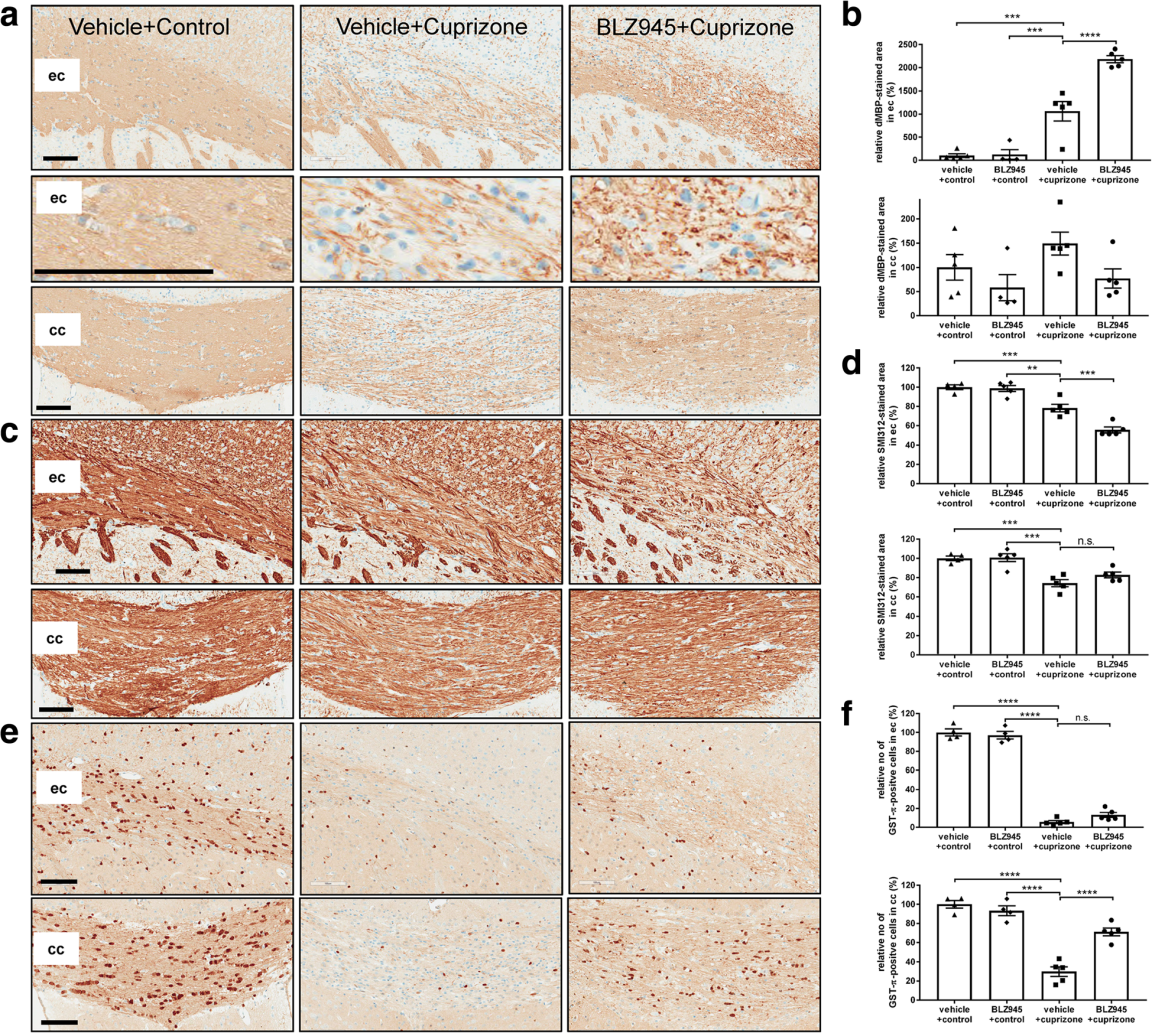
Prophylactic treatment with BLZ945 1 week before and during 5-week cuprizone intoxication revealed reduced oligodendrocyte numbers, appearance of myelin debris and axonal pathology in the external capsule but not in the corpus callosum. **a** Representative images from immunohistological stainings in the external capsule and corpus callosum detecting debris of myelin basic protein (dMBP). Myelin debris were obvious in the external capsule with BLZ945 and cuprizone treatment while no obvious change could be observed in the corpus callosum. **b** Corresponding quantitative analysis of the immunohistochemistry for dMBP-positive area in the corpus callosum and external capsule. Please note: The threshold for the staining of the control groups were below the image analysis parameters used. Values were normalized to those of control vehicle mice. **c** Representative images from immunohistological stainings in the external capsule and corpus callosum detecting neurofilament (SMI312) for the different treatment groups at week 5. Loss of axonal neurofilaments was obvious in the external capsule with BLZ945 and cuprizone treatment while no change could be observed in the corpus callosum. **d** Corresponding quantitative analysis of the immunohistochemistry for SMI312-positive area in the corpus callosum and external capsule. **e** Representative images from immunohistological stainings in the external capsule and corpus callosum detecting mature oligodendrocytes (GST-π) for the different treatment groups at week 5. Reduced oligodendrocyte numbers were observed in the external capsule while an increase in the corpus callosum was obvious with BLZ945 and cuprizone treatment. **f** Corresponding quantitative analysis of the immunohistochemistry for GST-π-positive soma numbers in the corpus callosum and external capsule. Values were normalized to those of control vehicle mice. Group sizes: for all treatment groups n = 4–5. Data are shown as means±SEM. Scale bars: 100 μm. Statistics: Turkey’s multiple comparison test one-way ANOVA **: *p* < 0.01, ***: *p* < 0.001, ****: *p* < 0.0001, n.s.: not significant), cpz: cuprizone, cc: corpus callosum, ec: external capsule

To analyze the involvement of microglia in the cuprizone model we depleted microglia before cuprizone administration. For this, mice were treated for 1 week before as well as during the cuprizone intoxication period with a high dose of BLZ945 (169 mg/kg p.o. qd) (Fig. 5a). The myelination status was assessed by longitudinal MRI (Fig. 5 and Additional file 1: Figure S10) and subsequent histology (Figs. 6 and 7 and Additional file 1: Figure S11, S12). Animals treated for 5 weeks with BLZ945 and cuprizone showed differential effects at the MRI in the corpus callosum and external capsule (Fig. 5b, see Fig. 5c for the region-of-interests used for MRI quantification). The external capsule displayed the expected demyelination as observed by increased MRI signal (Fig. 5b, d, green arrows). On contrary, in mice receiving both BLZ945 and cuprizone the corpus callosum did only show mild demyelination as suggested by the significantly lower MRI signal in comparison to that in the corpus callosum of animals from the cuprizone+vehicle group (Fig. 5b, d, red arrow). A slight but significant regional difference of MTR was obvious in the cuprizone group treated with BLZ945, but not the vehicle+cuprizone group (Fig. 5e). The MTR for the corpus callosum and external capsule was increased in BLZ945- compared to vehicle-treated mice. This increase in MTR (Fig. 5e) was even higher in the corpus callosum than the in external capsule in BLZ945-treated mice. These MRI effects were only obvious at week 5 (Additional file 1: Figure S10).

In analogy to the MRI observations, differential regional effects were also observed for demyelination and Iba1-positive microglia numbers after 5 weeks BLZ945 + cuprizone treatment (Fig. 6). At this time point, vehicle mice treated with cuprizone displayed again the expected demyelination (LFB) and increase of microglia (Iba1) in the corpus callosum and external capsule (Fig. 6, red and green arrows, respectively). However, mice with BLZ945 + cuprizone treatment for 5 weeks displayed in the corpus callosum a substantial amount of remaining myelin, but a reduction of Iba1-positive microglia (Fig. 6, red arrows). Furthermore, according to the increased presence of myelin, significantly more ODs in the corpus callosum could be detected (Fig. 7e, f). No difference of SMI312-positive axon fibers was observed in corpus callosum in mice treated with BLZ945 + cuprizone (Fig. 7c, d). Contrasting observations could be seen in the external capsule. Iba1-positive microglia were only slightly affected by the CSF1R kinase inhibition (Fig. 6c, d, green arrows), but a reduction of myelin (LFB) could be observed in the external capsule (Fig. 6a, d, green arrows). However, the external capsule displayed an enhanced presence of myelin debris (Fig. 7a, b) which was accompanied by reduced axonal fibers as detected by SMI312 (Fig. 7c, d) in mice treated with BLZ945 + cuprizone. In the cortex similar observations, enhanced myelin debris, axonal pathology and reduced NeuN-positive cells in the BLZ945 + cuprizone treatment group could be detected (Additional file 1: Figure S11). Finally, the external capsule displayed a similar reduction of ODs in vehicle+cuprizone and BLZ945 + cuprizone treated mice (Fig. 7e, f) correlating with the absence of myelin in this region. Again, as for the therapeutic BLZ945 treatment (Additional file 1: Figure S6c), in mice treated prophylactically with BLZ945 in the cuprizone model a dramatic reduction in NG2-positive cells was obvious (data not shown). Important to note, besides reducing microglia and NG2-positive cells there was no change in any of the analyzed parameters in mice treated for 6 weeks with BLZ945 alone without cuprizone intoxication. GFAP-positive astrocytes and the expression of the lysosomal marker Lamp-1 (CD107a) were increased during cuprizone treatment, but both were indifferent between the vehicle+cuprizone and BLZ9459 + cuprizone groups (Additional file 1: Figure S12). The similar increase of Lamp-1 in the external capsule in both treatment groups indicates that microglia are reacting similar to the intoxication but are in their function somehow impaired as myelin was not properly cleared and thus, could be detected as myelin debris. Similar microglia dysfunction in the same brain region could also be observed in MRI scans from TREM2 (triggering receptor expressed on myeloid cells 2 protein) knock-out animals treated for 5 weeks with cuprizone revealing as well increased myelin debris in the external capsule (Additional file 1: Figure S13).

In summary, prophylactic BLZ945 treatment before and during 5-week cuprizone intoxication reduced microglia in specific brain regions, prevented demyelination in the corpus callosum while myelin debris build up due to functionally impaired microglia could be observed in the external capsule.

## Discussion

In this study we pharmacologically depleted microglia therapeutically and prophylactically to investigate the contribution of microglia on de- and re-myelination processes in the cuprizone model. Cuprizone intoxication is a widely accepted experimental model to investigate MS-related pathology, characterized by a direct degeneration of mature oligodendrocytes (ODs, for review see [22, 50, 57]). We used the CSF1 receptor kinase inhibitor BLZ945 that has been shown previously to alter macrophage polarization and block glioma progression in preclinical models of glioblastomas [41] as well as to control myelin homeostasis in adult mouse brain [17]. We could show that BLZ945 dose-dependently depleted microglia in the central nervous system after 5 days of daily treatment consistent to a report by others that used a different CSF1R kinase inhibitor [10]. After inhibitor removal microglia repopulate readily and more importantly show unaltered function without any apparent adverse effects [9]. A complete absence of the CSF1 receptor on the contrary is detrimental for proper brain development [11].

The 2-week therapeutic treatment of BLZ945 in the 5-week cuprizone model showed a beneficial effect in the non-invasive longitudinal MRI signal intensity measurements as well as subsequent histology analysis on myelin level and OD numbers. MTR changes in these areas were not considered here, because of the small magnitude (≤2%) of MTR reductions in the cortex and striatum following the 5-week cuprizone intoxication period. The beneficial effect of BLZ945 was brain region-specific, with only the cortex and striatum showing increased remyelination whereas the effect was absent in the corpus callosum and external capsule. Other brain areas have not been analyzed in this study. The extent of beneficial effect obtained here was similar to enhanced remyelination in the cuprizone model described elsewhere [27] for therapeutic treatment with clemastine, a compound that is currently being tested in the clinic [14]. Furthermore, this differential brain region effect on myelin was also observed on microglia/astrocyte numbers as well as on the microglia activation status. In cortex and striatum the BLZ945-induced microglia reduction was much more pronounced than that observed in the corpus callosum and external capsule; the reduction of microglia was even below control levels in cortex and striatum. In contrast, the astrocytes were even further increased in cortex and striatum whereas this increase was only minimal in corpus callosum and external capsule. Similarly, morphological parameters characteristic of microglia activation were mostly increased in cortex and striatum, whereas there was no change or even a reduction of these parameters in corpus callosum and external capsule. This indicates that microglia numbers and morphology is brain region specifically altered, with subsequent consequences on myelination processes.

Astrocytes and microglia are known to play an important role in myelination in the cuprizone model (for review see [15]). Peripheral lipopolysaccharide (LPS) treatment in the cuprizone model delayed demyelination and enhanced remyelination by inhibiting microglia but enhancing oligondrocyte proliferation [47]. It has been also shown that astrocytes are needed to recruit microglia for clearing myelin debris and initiate repair [46]. Additionally, proper microglia function for clearing myelin debris is needed for efficient remyelination. It has been demonstrated through cuprizone intoxication that mice lacking the triggering receptor expressed on myeloid cells 2 (TREM2) and CX3CR1 microglia were not able to properly clear myelin and thus inefficiently remyelinated upon cuprizone removal [4, 26, 40]. Others have shown that enhancing microglia phagocytosis by inhibiting soluble TNF enhances remyelination [21]. Furthermore, laquinimod and quetiapine have been shown, via different mode of actions, to reduce microglia activation in the cuprizone model and promote myelin repair [2, 24, 52]. Microglia and astrocytes express different markers, have different polarization status (e.g. M1/M2 and A1/A2 classification) and functions during the course of the neuroinflammatory response to an insult [13, 28]. Despite the fact that M1/M2 phenotype classification for microglia has been challenged [42], as the boundaries between pro-inflammatory M1 and pro-resolution M2 microglia are not so well defined, it has been shown that M2 microglia promote oligodendrogenesis and thus shows enhanced remyelination [32, 55]. Thus, it might be conceivable that BLZ945 treatment alters the polarization of microglia/macrophages [41]. In our study the microglia depletion was not complete and the remaining microglia might have adopted an activation status that could promote myelin clearance, enhanced recruitment of astrocytes and increased OD proliferation and maturation. A thorough single-cell or cell population RNAseq expression analysis would be needed to investigate the microglia phenotype after BLZ945 treatment in the cuprizone model. Important to note, in human brain differences have been observed in the dynamics of OD generation between white and gray matter [54], a factor that could have also contributed to the differences in BLZ945 efficacy between cortex/striatum and corpus callosum/external capsule observed here. Furthermore, brain region-specific effects have been reported with chemokine knockout mice, showing beneficial effects in cortex but not corpus callosum [20], indicating that region-specific pathophysiological processes do exist. It has been shown that microglia are essential for the development and homeostasis of oligodendrocyte precursor cell (OPCs) [17]. We also could observe that BLZ945 treatment substantially reduced NG2-positive OPCs. Microglia seem to be essential in OPC maintenance as OPCs do not express CSF1R [17]. Nevertheless, mature ODs are still being generated and are even enhanced upon therapeutic BLZ945 treatment. It seems that OD numbers and function profit from microglia depletion. It can be speculated that in our model ODs are being generated by NG2-negative OPCs/pre-mature ODs or other unknown precursor cell populations. It has been shown that BLZ945 treatment induced an increase in GFP-positive/NG2-negative cells with the Sox10 promoter-driven inducible GFP reporter mouse [17], indicating an enhanced maturation of ODs or prompt differentiation of OPCs upon CSF1R pathway inhibition. Similar to Hagemeyer et al. [17] we also did not observe any myelination, axonal fiber and OD differences in the corpus callosum/external capsule after 6 weeks high dose BLZ945 treatment without cuprizone. Importantly, after BLZ945 removal OPCs similar to microglia seem to reappear [17].

Our data suggest that the microglia populations in the corpus callosum and the external capsule differed extensively in their behavior during prophylactic BLZ945 treatment in the cuprizone model. When BLZ945 was given prophylactically before and during cuprizone intoxication microglia in the external capsule became insensitive towards CSF1R kinase inhibition resulting in a functional impairment. It has been reported that heterozygous CSF1R knock-out mice also have abnormal myelination and aberrant activation of microglia [6]. This is similar to data observed in TREM2 (triggering receptor expressed on myeloid cells 2 protein) knock-out animals where microglia impairment could be observed by us and others [4, 40]. TREM2 and CSF1R presumably signal via the same adapter protein DAP12 [37] and when dysfunctional contributes to chronic neurodegeneration in humans. Deficiency in TREM2 leads to Nasu-Hakola disease and patients with partial loss-of-function mutations in CSF1R suffer from hereditary diffuse leukoencephalopathy with spheroids (see reviews [34, 53]). This indicates that the CSF1R and TREM2 pathway may converge and even interact to exert similar downstream events. At week 5, there was a discrepancy between the MRI readouts at the level of the external capsule of cuprizone-challenged mice: whereas the signal intensities were approximately the same for the BLZ945 and vehicle groups, consistent with the similar levels of demyelination in both groups evidenced by histology, the MTR was significantly increased in BLZ945- compared to vehicle-treated animals. However, increased myelin debris was observed by histology in the external capsule of mice that had received BLZ945, suggesting that myelin debris contributed to MTR. These observations are consistent with earlier work showing that OD ablation resulted only in minor decreases in MTR due to inefficient removal of myelin debris [33]. In the corpus callosum on the contrary, for BLZ945 given prophylactically, microglia were substantially reduced, comparable to the situation in the therapeutic BLZ945 treatment mode, and additionally microglia functionality was altered, leading to reduced demyelination, no accumulation of myelin debris and absence of axonal damage. This is in agreement with the reduced MRI signal and the increased MTR measured in the corpus callosum of cuprizone challenged animals receiving BLZ945 prophylactically. Therefore, a discrepancy between MRI signal and MTR might thus indicate poor myelin debris removal in a given area, pointing to the importance of assessing both parameters.

Microglia can also exert pro-inflammatory neurotoxic function in chronic neurodegenerative diseases, damaging neurons, synapses, neuronal processes directly or indirectly by helping spreading of toxic proteins. It has been shown that CSF1R kinase inhibition in animal models of Alzheimer’s disease (AD), amyotrophic lateral sclerosis (ALS), brain lesions, tauopathies and catatonia of neuropsychiatric diseases prevented or functionally improved disease progression [1, 8, 19, 30, 36, 43]. Similarly, mice lacking the chemokine Cxcl10 showed a reduction of microglia activation and as a consequence a reduced demyelination and axonal pathology [7]. Brain areas with the strongest microglia depletion by BLZ945 also displayed enhanced myelination and OD numbers. This implicates that a reduction of microglia is beneficial for the myelination processes exerted by remaining ODs. The exact mechanisms by which microglia are inhibiting mature OD myelination remains to be elucidated. Important to note that in an experimental autoimmune encephalomyelitis (EAE) model therapeutic treatment of BLZ945 did not change disease progression despite a significant reduction of microglia cells in the spinal cord gray matter. However, microglia were not reduced in the cortex, but showed enhanced proliferation indicating that peripheral immune cells in the EAE model do alter microglia response towards BLZ945. Further studies are needed to characterize the phenotype of microglia in the EAE model. Additionally, combination therapies in the EAE model for modulating peripheral as well as central inflammatory processes may shed more light on the contribution of microglia to the pathology. Furthermore, combining cuprizone and EAE models could prove to have good translational value for studying inflammatory lesions in the forebrain [44, 45]. Nevertheless, our results indicate that microglia modulation via the CSF1R pathway seems not to play a crucial role in modifying the disease progression in the EAE model.

Taken together, the myelination processes can be positively modulated by reducing microglia and enhancing astrocytes and thereby increasing and/or preserve ODs and, finally, myelination. As this is brain region-specific and depends on disease state the timing on the intervention is crucial. Our results show for the first time beneficial effects on myelination events in the cuprizone model by CSF1R kinase inhibition. Thus, microglia-modulating mechanisms could be potentially pursued as new short-term treatment paradigms for initiating and enhancing myelination processes in combination with standard-of-care medication in MS.

## Additional file

Additional file 1: Figure S1-S14. **Figure S1**. 5-week cuprizone intoxication leads to demyelination and neuroinflammation in the corpus callosum when compared to healthy control. **Figure S2**. MRI reliably detects de- and re-myelination events in the cuprizone model in the corpus callosum/external capsule (cc+ec), correlating with myelin and oligodendrocyte histology. **Figure S3**. BLZ945 dose-response and time-course on microglia depletion and activation in the brain. **Figure S4**. BLZ945 dose-response on microglia-specific gene expression and microglia depletion in the spinal cord. **Figure S5**. Longitudinal MRI measurements of a 2-week therapeutic treatment with BLZ945 (169 mg/kg p.o., qd) after 5-week cuprizone intoxication. **Figure S6**. A 2-week therapeutic treatment with BLZ945 after 5-week cuprizone intoxication period enhanced remyelination but depleted NG2-positive oligodendrocyte precursor cells. **Figure S7**. A 2-week therapeutic treatment with BLZ945 after a 5-week cuprizone intoxication period changed microglia morphology. **Figure S8**. A 2-week therapeutic treatment with BLZ945 after a 5-week cuprizone intoxication period enhanced astrogliosis. **Figure S9**. Therapeutic BLZ945 treatment in experimental autoimmune encephalomyelitis (EAE) mice did not alter disease progression. Microglia in the spinal cord were reduced, whereas in the cortex no reduction, but enhanced microglia proliferation could be observed. **Figure S10**. Longitudinal MRI measurements of prophylactic treatment with BLZ945 1 week before and during the 5-week cuprizone intoxication. **Figure S11**. Prophylactic treatment with BLZ945 before and during cuprizone intoxication led to axonal pathology, myelin debris and reduced NeuN-positive cells in cortex. **Figure S12**. Prophylactic treatment with BLZ945 before and during cuprizone intoxication showed similar levels of astrocytosis as well as increase in LAMP1 in external capsule (ec) and corpus callosum (cc). **Figure S13.** 5-week cuprizone intoxication in TREM2 knock-out mice led to enhanced pathology especially in the external capsule. **Figure S14**. Quantitative image analysis of microglia/astrocyte numbers and morphology of stained Iba1 and GFAP brain sections. (PDF 13675 kb)

## References

[1] Asai H, Ikezu S, Tsunoda S, Medalla M, Luebke J, Haydar T, Wolozin B, Butovsky O, Kügler S, Ikezu T (2015). Depletion of microglia and inhibition of exosome synthesis halt tau propagation. Nat Neurosci.

[2] Brück W, Pförtner R, Pham T, Zhang J, Hayardeny L, Piryatinsky V, Hanisch U-K, Regen T, van Rossum D, Brakelmann L, Hagemeier K, Kuhlmann T, Stadelmann C, John GR, Kramann N, Wegner C (2012). Reduced astrocytic NF-κB activation by laquinimod protects from cuprizone-induced demyelination. Acta Neuropathol.

[3] Calabrese M, Magliozzi R, Ciccarelli O, Geurts JJG, Reynolds R, Martin R (2015). Exploring the origins of grey matter damage in multiple sclerosis. Nat Rev Neurosci.

[4] Cantoni C, Bollman B, Licastro D, Xie M, Mikesell R, Schmidt R, Yuede CM, Galimberti D, Olivecrona G, Klein RS, Cross AH, Otero K, Piccio L (2015) TREM2 regulates microglial cell activation in response to demyelination in vivo. Acta Neuropathol. doi: 10.1007/s00401-015-1388-1 10.1007/s00401-015-1388-1 PMC4667728 25631124

[5] Chandran P, Upadhyay J, Markosyan S, Lisowski a, Buck W, Chin C-L, Fox G, Luo F, Day M (2012). Magnetic resonance imaging and histological evidence for the blockade of cuprizone-induced demyelination in C57BL/6 mice. Neuroscience.

[6] Chitu V, Gokhan S, Gulinello M, Branch CA, Patil M, Basu R, Stoddart C, Mehler MF, Richard Stanley E (2015). Phenotypic characterization of a Csf1r haploinsufficient mouse model of adult-onset leukodystrophy with axonal spheroids and pigmented glia (ALSP). Neurobiol Dis.

[7] Clarner T, Janssen K, Nellessen L, Stangel M, Skripuletz T, Krauspe B, Hess F-M, Denecke B, Beutner C, Linnartz-Gerlach B, Neumann H, Vallières L, Amor S, Ohl K, Tenbrock K, Beyer C, Kipp M (2015) CXCL10 Triggers Early Microglial Activation in the Cuprizone Model. J Immunol. doi: 10.4049/jimmunol.1401459 10.4049/jimmunol.1401459 25725102

[8] Dagher NN, Najafi AR, Kayala KMN, Elmore MRP, White TE, Medeiros R, West BL, Green KN (2015). Colony-stimulating factor 1 receptor inhibition prevents microglial plaque association and improves cognition in 3xTg-AD mice. J Neuroinflammation.

[9] Elmore MRP, Lee RJ, West BL, Green KN (2015). Characterizing newly repopulated microglia in the adult mouse: impacts on animal behavior, cell morphology, and neuroinflammation. PLoS One.

[10] Elmore MRP, Najafi AR, M a K, Dagher NN, Spangenberg EE, Ra R, Kitazawa M, Matusow B, Nguyen H, West BL, Green KN (2014). Colony-stimulating factor 1 receptor signaling is necessary for microglia viability, unmasking a microglia progenitor cell in the adult brain. Neuron.

[11] Erblich B, Zhu L, Etgen AM, Dobrenis K, Pollard JW (2011). Absence of colony stimulation factor-1 receptor results in loss of microglia, disrupted brain development and olfactory deficits. PLoS One.

[12] Fjær S, Bø L, Lundervold A, Myhr K-M, Pavlin T, Torkildsen O, Wergeland S (2013). Deep gray matter demyelination detected by magnetization transfer ratio in the cuprizone model. PLoS One.

[13] Franco R, Fernández-Suárez D (2015). Alternatively activated microglia and macrophages in the central nervous system. Prog Neurobiol.

[14] Green AJ, Gelfand JM, Cree BA, Bevan C, Boscardin WJ, Mei F, Inman J, Arnow S, Devereux M, Abounasr A, Nobuta H, Zhu A, Friessen M, Gerona R, von Büdingen HC, Henry RG, Hauser SL, Chan JR (2017) Clemastine fumarate as a remyelinating therapy for multiple sclerosis (ReBUILD): A randomised, controlled, double-blind, crossover trial. Lancet. 10.1016/S0140-6736(17)32346-2 10.1016/S0140-6736(17)32346-2 29029896

[15] Gudi V, Gingele S, Skripuletz T, Stangel M (2014). Glial response during cuprizone-induced de- and remyelination in the CNS: lessons learned. Front Cell Neurosci.

[16] Haase A, Matthaei D, Hänicke W, Frahm J (1986). Dynamic digital subtraction imaging using fast low-angle shot MR movie sequence. Radiology.

[17] Hagemeyer N, Hanft K-M, Akriditou M-A, Unger N, Park ES, Stanley ER, Staszewski O, Dimou L, Prinz M (2017) Microglia contribute to normal myelinogenesis and to oligodendrocyte progenitor maintenance during adulthood. Acta Neuropathol. 10.1007/s00401-017-1747-1 10.1007/s00401-017-1747-1 PMC5951721 28685323

[18] Hennig J, Nauerth A, Friedburg H (1986). RARE imaging: a fast imaging method for clinical MR. Magn Reson Med.

[19] Janova H, Arinrad S, Balmuth E, Mitjans M, Hertel J, Habes M, Bittner RA, Pan H, Goebbels S, Begemann M, Gerwig UC, Langner S, Werner HB, Kittel-Schneider S, Homuth G, Davatzikos C, Völzke H, West BL, Reif A, Grabe HJ, Boretius S, Ehrenreich H, Nave K-A (2017) Microglia ablation alleviates myelin-associated catatonic signs in mice. J Clin Invest:1–12. 10.1172/JCI97032 10.1172/JCI97032 PMC5785265 29252214

[20] Janssen K, Rickert M, Clarner T, Beyer C, Kipp M (2015) Absence of CCL2 and CCL3 ameliorates central nervous system Grey matter but not white matter Demyelination in the presence of an intact blood–brain barrier. Mol Neurobiol:1551–1564. 10.1007/s12035-015-9113-6 10.1007/s12035-015-9113-6 25663168

[21] Karamita M, Barnum C, Möbius W, Tansey MG, Szymkowski DE, Lassmann H, Probert L (2017) Therapeutic inhibition of soluble brain TNF promotes remyelination by increasing myelin phagocytosis by microglia. JCI Insight. 10.1172/jci.insight.87455 10.1172/jci.insight.87455 PMC5396518 28422748

[22] Kipp M, Clarner T, Dang J, Copray S, Beyer C (2009). The cuprizone animal model: new insights into an old story. Acta Neuropathol.

[23] Klein D, Patzko I, Schreiber D, van Hauwermeiren A, Baier M, Groh J, West BL, Martini R (2015). Targeting the CSF-1 receptor alleviates two forms of Charcot-Marie-tooth disease in mice. Glia.

[24] Kramann N, Menken L, Hayardeny L, Hanisch U-K, Brück W (2016). Laquinimod prevents cuprizone-induced demyelination independent of toll-like receptor signaling. Neurol Neuroimmunol Neuroinflamm.

[25] J a K, Jin Y, Walles M, Pfaar U, Sutton J, Wiesmann M, Graf D, Pflimlin-Fritschy V, Wolf T, Camenisch G, Swart P (2015). Phenotypic and metabolic investigation of a CSF-1R kinase receptor inhibitor (BLZ945) and its pharmacologically active metabolite. Xenobiotica.

[26] Lampron A, Larochelle A, Laflamme N, Préfontaine P, Plante M-M, Sánchez MG, Yong VW, Stys PK, Tremblay M-È, Rivest S (2015). Inefficient clearance of myelin debris by microglia impairs remyelinating processes. J Exp Med.

[27] Li Z, He Y, Fan S, Sun B (2015). Clemastine rescues behavioral changes and enhances remyelination in the cuprizone mouse model of demyelination. Neurosci Bull.

[28] Liddelow SA, Guttenplan KA, Clarke LE, Bennett FC, Bohlen CJ, Schirmer L, Bennett ML, Münch AE, Chung W-S, Peterson TC, Wilton DK, Frouin A, Napier BA, Panicker N, Kumar M, Buckwalter MS, Rowitch DH, Dawson VL, Dawson TM, Stevens B, Barres BA (2017) Neurotoxic reactive astrocytes are induced by activated microglia. Nature. 10.1038/nature21029 10.1038/nature21029 PMC5404890 28099414

[29] Luo J, Elwood F, Britschgi M, Villeda S, Zhang H, Ding Z, Zhu L, Alabsi H, Getachew R, Narasimhan R, Wabl R, Fainberg N, James ML, Wong G, Relton J, Gambhir SS, Pollard JW, Wyss-Coray T (2013). Colony-stimulating factor 1 receptor (CSF1R) signaling in injured neurons facilitates protection and survival. J Exp Med.

[30] Martínez-Muriana A, Mancuso R, Francos-Quijorna I, Olmos-Alonso A, Osta R, Perry VH, Navarro X, Gomez-Nicola D, López-Vales R (2016). CSF1R blockade slows the progression of amyotrophic lateral sclerosis by reducing microgliosis and invasion of macrophages into peripheral nerves. Sci Rep.

[31] Merkler D, Boretius S, Stadelmann C, Ernsting T, Michaelis T, Frahm J, Brück W (2005). Multicontrast MRI of remyelination in the central nervous system. NMR Biomed.

[32] Miron VE, Boyd A, Zhao J-W, Yuen TJ, Ruckh JM, Shadrach JL, van Wijngaarden P, Wagers AJ, Williams A, Franklin RJM, ffrench-Constant C (2013) M2 microglia and macrophages drive oligodendrocyte differentiation during CNS remyelination. Nat Neurosci 16:1211–1218. doi: 10.1038/nn.3469 10.1038/nn.3469 PMC3977045 23872599

[33] Mueggler T, Pohl H, Baltes C, Riethmacher D, Suter U, Rudin M (2012). MRI signature in a novel mouse model of genetically induced adult oligodendrocyte cell death. NeuroImage.

[34] Neumann H, Daly MJ (2013). Variant TREM2 as risk factor for Alzheimer’s disease. N Engl J Med.

[35] Nylander A, Hafler DA (2012). Multiple sclerosis. J Clin Invest.

[36] Olmos-Alonso A, Schetters STT, Sri S, Askew K, Mancuso R, Vargas-Caballero M, Holscher C, Perry VH, Gomez-Nicola D (2016). Pharmacological targeting of CSF1R inhibits microglial proliferation and prevents the progression of Alzheimer’s-like pathology. Brain.

[37] Otero K, Turnbull IR, Poliani PL, Vermi W, Cerutti E, Aoshi T, Tassi I, Takai T, Stanley SL, Miller M, Shaw AS, Colonna M (2009). Macrophage colony-stimulating factor induces the proliferation and survival of macrophages via a pathway involving DAP12 and beta-catenin. Nat Immunol.

[38] Perry VH (2016). Microglia. Microbiol Spectr.

[39] Petković F, Campbell IL, Gonzalez B, Castellano B (2016) Astrocyte-targeted production of interleukin-6 reduces astroglial and microglial activation in the cuprizone demyelination model: implications for myelin clearance and oligodendrocyte maturation. Glia. 10.1002/glia.23043 10.1002/glia.23043 27535761

[40] Poliani PL, Wang Y, Fontana E, Robinette ML, Yamanishi Y, Gilfillan S, Colonna M (2015). TREM2 sustains microglial expansion during aging and response to demyelination. J Clin Invest.

[41] Pyonteck SM, Akkari L, Schuhmacher AJ, Bowman RL, Sevenich L, Quail DF, Olson OC, Quick ML, Huse JT, Teijeiro V, Setty M, Leslie CS, Oei Y, Pedraza A, Zhang J, Brennan CW, Sutton JC, Holland EC, Daniel D, Joyce JA (2013). CSF-1R inhibition alters macrophage polarization and blocks glioma progression. Nat Med.

[42] Ransohoff RM (2016). A polarizing question: do M1 and M2 microglia exist?. Nat Neurosci.

[43] Rice R a, Spangenberg EE, Yamate-Morgan H, Lee RJ, RPS A, Hernandez MX, Tenner a J, West BL, Green KN (2015). Elimination of microglia improves functional outcomes following extensive neuronal loss in the hippocampus. J Neurosci.

[44] Rüther BJ, Scheld M, Dreymueller D, Clarner T, Kress E, Brandenburg L-O, Swartenbroekx T, Hoornaert C, Ponsaerts P, Fallier-Becker P, Beyer C, Rohr SO, Schmitz C, Chrzanowski U, Hochstrasser T, Nyamoya S, Kipp M (2017). Combination of cuprizone and experimental autoimmune encephalomyelitis to study inflammatory brain lesion formation and progression. Glia.

[45] Scheld M, Ruther BJ, Grosse-Veldmann R, Ohl K, Tenbrock K, Dreymuller D, Fallier-Becker P, Zendedel A, Beyer C, Clarner T, Kipp M (2016). Neurodegeneration triggers peripheral immune cell recruitment into the forebrain. J Neurosci.

[46] Skripuletz T, Hackstette D, Bauer K, Gudi V, Pul R, Voss E, Berger K, Kipp M, Baumgärtner W, Stangel M (2013). Astrocytes regulate myelin clearance through recruitment of microglia during cuprizone-induced demyelination. Brain.

[47] Skripuletz T, Miller E, Grote L, Gudi V, Pul R, Voss E, Skuljec J, Moharregh-Khiabani D, Trebst C, Stangel M (2011). Lipopolysaccharide delays demyelination and promotes oligodendrocyte precursor proliferation in the central nervous system. Brain Behav Immun.

[48] Spangenberg EE, Lee RJ, Najafi AR, Rice R a, Elmore MRP, Blurton-Jones M, West BL, Green KN (2016) Eliminating microglia in Alzheimer’s mice prevents neuronal loss without modulating amyloid-β pathology. Brain 1–17. 10.1093/brain/aww016 10.1093/brain/aww016 PMC5006229 26921617

[49] Thiessen JD, Zhang Y, Zhang H, Wang L, Buist R, Del Bigio MR, Kong J, Li X-M, Martin M (2013). Quantitative MRI and ultrastructural examination of the cuprizone mouse model of demyelination. NMR Biomed.

[50] Torkildsen O, Brunborg LA, Myhr K-M, Bø L (2008). The cuprizone model for demyelination. Acta Neurol Scand Suppl.

[51] Turati L, Moscatelli M, Mastropietro A, Dowell NG, Zucca I, Erbetta A, Cordiglieri C, Brenna G, Bianchi B, Mantegazza R, Cercignani M, Baggi F, Minati L (2015). In vivo quantitative magnetization transfer imaging correlates with histology during de- and remyelination in cuprizone-treated mice. NMR Biomed.

[52] Wang H, Liu S, Tian Y, Wu X, He Y, Li C, Namaka M, Kong J, Li H, Xiao L (2015). Quetiapine inhibits Microglial activation by neutralizing abnormal STIM1-mediated intercellular calcium homeostasis and promotes myelin repair in a Cuprizone-induced mouse model of Demyelination. Front Cell Neurosci.

[53] Yaghmoor F, Noorsaeed A, Alsaggaf S, Aljohani W, Scholtzova H, Boutajangout A, Wisniewski T (2014) The role of TREM2 in Alzheimer’s disease and other neurological disorders. J Alzheimer’s Dis Park. 10.4172/2161-0460.1000160 10.4172/2161-0460.1000160 PMC4317331 25664220

[54] Yeung MSYSY, Zdunek S, Bergmann O, Bernard S, Salehpour M, Alkass K, Perl S, Tisdale J, Possnert G, Brundin L, Druid H, Frisén J (2014). Dynamics of Oligodendrocyte generation and Myelination in the human brain. Cell.

[55] Yuan J, Ge H, Liu W, Zhu H, Chen Y, Zhang X, Yang Y, Yin Y, Chen W, Wu W, Yang Y, Lin J (2017). M2 microglia promotes neurogenesis and oligodendrogenesis from neural stem/progenitor cells via the PPARγ signaling pathway. Oncotarget.

[56] Zaaraoui W, Deloire M, Merle M, Girard C, Raffard G, Biran M, Inglese M, Petry KG, Gonen O, Brochet B, Franconi J-M, Dousset V (2008). Monitoring demyelination and remyelination by magnetization transfer imaging in the mouse brain at 9.4 T. MAGMA.

[57] Zendedel A, Beyer C, Kipp M (2013). Cuprizone-induced demyelination as a tool to study remyelination and axonal protection. J Mol Neurosci.

