# Supplementary material for: Brain region-specific enhancement of remyelination and prevention of demyelination by the CSF1R kinase inhibitor BLZ945
Source: Acta Neuropathol Commun. 2018 Feb 15;6:9. doi: 10.1186/s40478-018-0510-8 (PMC5815182; doi:10.1186/s40478-018-0510-8)
Supplement: Supplementary file 1 — Figure S1. 5-week cuprizone intoxication leads to demyelination and neuroinflammation in the corpus callosum when compared to healthy control. Figure S2. MRI reliably detects de- and re-myelination events in the cuprizone model in the corpus callosum/external capsule (cc+ec), correlating with myelin and oligodendrocyte histology. Figure S3. BLZ945 dose-response and time-course on microglia depletion and activation in the brain. Figure S4. BLZ945 dose-response on microglia-specific gene expression and microglia depletion in the spinal cord. Figure S5. Longitudinal MRI measurements of a 2-week therapeutic treatment with BLZ945 (169 mg/kg p.o., qd) after 5-week cuprizone intoxication. Figure S6. A 2-week therapeutic treatment with BLZ945 after 5-week cuprizone intoxication period enhanced remyelination but depleted NG2-positive oligodendrocyte precursor cells. Figure S7. A 2-week therapeutic treatment with BLZ945 after a 5-week cuprizone intoxication period changed microglia morphology. Figure S8. A 2-week therapeutic treatment with BLZ945 after a 5-week cuprizone intoxication period enhanced astrogliosis. Figure S9. Therapeutic BLZ945 treatment in experimental autoimmune encephalomyelitis (EAE) mice did not alter disease progression. Microglia in the spinal cord were reduced, whereas in the cortex no reduction, but enhanced microglia proliferation could be observed. Figure S10. Longitudinal MRI measurements of prophylactic treatment with BLZ945 1 week before and during the 5-week cuprizone intoxication. Figure S11. Prophylactic treatment with BLZ945 before and during cuprizone intoxication led to axonal pathology, myelin debris and reduced NeuN-positive cells in cortex. Figure S12. Prophylactic treatment with BLZ945 before and during cuprizone intoxication showed similar levels of astrocytosis as well as increase in LAMP1 in external capsule (ec) and corpus callosum (cc). Figure S13. 5-week cuprizone intoxication in TREM2 knock-out mice led to enhanced pathology espec [file 40478_2018_510_MOESM1_ESM.pdf]

Additional file

Brain region-specific enhancement of remyelination and prevention of demyelination by the CSF1R kinase inhibitor BLZ945

Nicolau Beckmann[1], Elisa Giorgetti[1], Anna Neuhaus[2], Stefan Zurbruegg[2], Nathalie Accart[1], Paul Smith[3+], Julien Perdoux[3], Ludovic Perrot[4], Mark Nash[1], Sandrine Desrayaud[5], Peter Wipfli[5], Wilfried Frieauff[6], Derya R. Shimshek[2*]

[1]Muscoskeletal Disease Area, [2]Neuroscience, [3]Autoimmunity, Transplantation and Inflammation, [4]Global Scientific Operations, [5]PK Sciences, [6]Preclinical Safety, Novartis Institutes for BioMedical Research, Novartis Pharma AG, CH-4002 Basel, Switzerland

+current address: Incyte, 1801 Augustine Cut-off, Wilmington, DE 19803, USA

*corresponding author: derya.shimshek@novartis.com, +41795435384

Additional file 1

Figure S1. 5-week cuprizone intoxication leads to demyelination and neuroinflammation in the corpus callosum when compared to healthy control. Representative pictures from histological and immunohistological stainings from the corpus callosum after 5-week 0.2% cuprizone food and control food treatment of C57BL/6 mice detecting myelin by Luxol fast blue (LFB), microglia (Iba1) and astrocytes by glial fibrillary acidic protein (GFAP). Scale bars: 2 mm.

Figure S2. MRI reliably detects de- and re-myelination events in the cuprizone model in the corpus callosum/external capsule (cc+ec), correlating with myelin and oligodendrocyte histology. **a** MRI signal and magnetization transfer ratio (MTR) in the corpus callosum for the different treatment groups. Values were normalized to absolute values of the vehicle control group (normal food). **b** Quantitative analysis of the optical density (OD) analysis of Luxol fast blue (LFB) histological staining, of the immunohistochemistry for MOG and of GST-π positive cells in the cc+ec. Values were normalized to values in the control group. Group sizes: n= 6-7. Data is shown as mean±SEM. Statistics: Holm-Sidak's multiple comparisons test one-way ANOVA (*: $p<0.05$, **: $p<0.01$, ****: $p<0.0001$, n.s.: not significant).

Figure S3. BLZ945 dose-response and time-course on microglia depletion and activation in the brain. **a** Representative pictures from immunohistological stainings detecting the microglia marker Iba1 in the cortex for the different treatment groups. Groups consisted of mice treated for 5 days with vehicle or BLZ945 p.o., qd at 7, 20, 60, 100 and 169 mg/kg as indicated. Inset show higher magnification. Scale bars: 100 µm. **b** Quantitative analysis of the immunohistochemistry for Iba1-positive microglia numbers in the cortex normalized to control vehicle for the time-course of BLZ945. The time-course consisted of a 5-day treatment with 169 mg/kg BLZ945, p.o., qd and then recovery for 3 or 7 days. The time-course shows depletion of microglia after 5 days of BLZ945 treatment and a quick recovery within 3 days with full recovery after 7 days of treatment discontinuation. Furthermore, microglia activation (microglia soma and proximal processes area normalized to distal processes) is increased with BLZ945 treatment. Microglia in the recovery phase at 3 days are highly activated. **c** Quantitative analysis of the immunohistochemistry for Iba1-positive microglia numbers in the cortex normalized to control vehicle (means±sem, n=3 mice per group) for the dose-response of BLZ945. The dose-response of BLZ945 consisted of treatment with different doses of BLZ945 (7, 20, 60, 100, 169 mg/kg p.o., qd) for 5 days. BLZ945 showed dose-dependent microglia depletion after 5-day treatment. d Pharmacokinetic analysis of BLZ945 in blood and brain. The blood and brain BLZ945 levels were dose-dependent and showed a brain/blood ratio of 0.3-0.6 as indicated above the respective graphs. Group sizes: n= 2-3. Data is shown as mean±SEM. Statistics: Dunnett's multiple comparison test one-way ANOVA (*: $p<0.05$, **: $p<0.01$, ***: $p<0.001$, ****: $p<0.0001$).

Figure 4. BLZ945 dose-response on microglia-specific gene expression and microglia depletion in the spinal cord. **a** Representative pictures from immunohistological stainings detecting the microglia marker Iba1 in the spinal cord for the different treatment groups. Groups consisted of mice treated for 5 days with vehicle or BLZ945 p.o., qd

at 7, 20, 60, 100 and 169 mg/kg as indicated. Scale bar: 60 µm. **b** Quantitative analysis of gene transcriptions by qRT-PCR for microglia-specific genes. Values indicate the relative expression with respect to vehicle group (means±sem, for n=3 mice per group). Transmembrane protein 119 precursor (Tmem119), CX3C chemokine receptor 1 (fractalkine receptor, CX3CR1), colony stimulating factor 1 receptor (CSF1R), allograft inflammatory factor 1 (Aif1) and triggering receptor expressed on myeloid cells 2 (Trem2) were dose-dependently reduced by BLZ945 confirming microglia depletion. Statistics: Dunnett's multiple comparisons test one-way ANOVA (**: p<0.01, ***: p<0.001, ****: p<0.0001, n.s.: not significant).

Figure S5. Longitudinal MRI measurements of a 2-week therapeutic treatment with BLZ945 (169 mg/kg p.o., qd) after 5-week cuprizone intoxication. **a, b, c, d** MRI signal in cortex, striatum and corpus callosum (cc)+external capsule (ec) as well as MTR for in the cc+ec for the different treatment groups (means±sem). MRI signal and MTR were normalized to absolute values of the control group (normal food, vehicle treatment). Group sizes: n= 13-14. Data is shown as mean±SEM. Statistics: Holm-Sidak's multiple comparisons test one-way ANOVA (**: p<0.01, ***: p<0.001).

Figure S6. A 2-week therapeutic treatment with BLZ945 after 5-week cuprizone intoxication period enhanced remyelination but depleted NG2-positive oligodendrocyte precursor cells. **a** Representative overview pictures of a half-brain of a MBP (myelin basic protein) immunohistological staining for the different treatment groups. Scale bar: 1 mm. **b** Quantification of MBP in the cortex (M1 region specifically) detecting either MBP-stained area (normalized to region-of-interest) or integrated density (IntDen) measurement with threshold by ImageJ. **c** Representative pictures of a NG2 (nerve-glia antigen 2) immunohistological staining for the different treatment groups. Brain regions are indicated. cc/ec: corpus callosum/external capsule. Scale bar: 200 µm.

Figure S7. A 2-week therapeutic treatment with BLZ945 after a 5-week cuprizone intoxication period changed microglia morphology. **a, b, c** Quantitative analysis of the immunohistochemistry for Iba1-positive microglia in cortex, striatum and corpus callosum (cc)+external capsule (ec) for microglia size (microglia soma and proximal processes area normalized to microglia numbers), microglia form factor and microglia activation (microglia soma and proximal processes area normalized to distal process area). Group sizes: n=7. Data are shown as means±SEM. Statistics: Holm Sidak`s multiple comparison test one-way ANOVA (*: p<0.05, **: p<0.01, ***: p<0.001, ****: p<0.0001, n.s.: not significant).

Figure S8. A 2-week therapeutic treatment with BLZ945 after a 5-week cuprizone intoxication period enhanced astrogliosis. Representative overview pictures of ALDH1L1 (aldehyde dehydrogenase 1 family member L1) immunohistological staining. Scale bars: 1 mm.

Figure S9. Therapeutic BLZ945 treatment in experimental autoimmune encephalomyelitis (EAE) mice did not alter disease progression. Microglia in the spinal cord were reduced, whereas in the cortex no reduction, but enhanced microglia proliferation could be observed. **a** Evaluation of the clinical score after myelin oligodendrocyte glycoprotein peptide (MOG)-immunization revealed comparable disease progression, disease onset (defined by a

clinical score ≥ 1) and weight changes for all treatment groups. BLZ945 treatment started at 14 days post-immunization near a maximum clinical score and continued for 14 days (shaded area). Experiment ended at 28 days post-immunization. For all groups: n=10 mice. Data are shown as means±SEM. **b** Quantitative analysis of the immunohistochemistry for Iba1-positive microglia in the gray matter of the soinal cord and cortex for microglia numbers and microglia activation (microglia soma and proximal processes area normalized to distal process area). Group sizes: n=6-7. Data are shown as means±SEM. Statistics: Holm Sidak`s multiple comparison test one-way ANOVA (*: $p<0.05$). **c, d** Representative overview pictures of the spinal cord and higher magnification pictures of the gray matter spinal cord (**c**) and cortex (**d**) from immunohistological stainings detecting the microglia marker Iba1. Scale bars: 300 µm (overview pictures) and 100 µm (higher magnification). **e** Representative pictures of cortex from double-immunofluorescence stainings detecting the microglia marker Iba1 and the proliferation marker Ki67 in a BLZ945-treated EAE mouse.

Figure S10. Longitudinal MRI measurements of prophylactic treatment with BLZ945 1 week before and during the 5-week cuprizone intoxication. **a, b, c** MRI contrast (a.u.: absolute values) in corpus callosum and external capsule, cortex and striatum for the different treatment groups. Treatment groups: baseline (before cuprizone treatment, 1 week with and without BLZ945 treatment (p.o., qd, 169 mg/kg)), week3 (normal food or cuprizone 0.2% treatment in food for 3 weeks with and without BLZ945 treatment (p.o., qd, 169 mg/kg), respectively) and 5 weeks (normal food or cuprizone 0.2% treatment in food for 5 weeks with and without BLZ945 treatment (p.o., qd, 169 mg/kg), respectively). MRI contrast in corpus callosum and external capsule in BLZ945+cuprizone treated animals is similar at week3 and reduced at week5 compared to the vehicle+cuprizone treatment group. A similar effect was obvious in striatum whereas no difference was obvious in cortex. Group sizes: n=5. Data are shown as means±SEM. Statistics: Holm-Sidak's multiple comparison test two-way ANOVA (*: $p<0.05$, ****: $p<0.0001$).

Figure S11. Prophylactic treatment with BLZ945 before and during cuprizone intoxication led to axonal pathology, myelin debris and reduced NeuN-positive cells in cortex. **a, b, c** Representative pictures from immunohistological stainings for neurofilament (SMI312), debris of myelin basic protein (dMBP) and NeuN for neurons. Mice were treated with BLZ945 (p.o., qd, 169 mg/kg) or vehicle (1 week) before and during 0.2% cuprizone intoxication (5 weeks). Scale bars: 100 µm. d Quantitative analysis of the immunohistochemistry for SMI312- and dMBP-positive stained area and NeuN-positive soma numbers in the cortex (M1). Group sizes: n=4-5. Data are shown as means±SEM. Statistics: Holm-Sidak's multiple comparison test two-way ANOVA (*: $p<0.05$, **: $p<0.01$, ****: $p<0.0001$).

Figure S12. Prophylactic treatment with BLZ945 before and during cuprizone intoxication showed similar levels of astrocytosis as well as increase in LAMP1 in external capsule (ec) and corpus callosum (cc). Representative pictures from immunohistological stainings for GFAP (glial fibrillary acidic protein) and CD107a (LAMP-1). Mice were treated with BLZ945 (p.o., qd, 169 mg/kg) or vehicle (1 week) before and during 0.2% cuprizone intoxication (5 weeks). Scale bars: 2 mm (overview pictures) and 200 µm.

Figure S13. 5-week cuprizone intoxication in TREM2 knock-out mice led to enhanced pathology especially in the external capsule. Representative MRI brain images and pictures from immunohistological stainings detecting dMBP from external capsule of wildtype (WT) and TREM2 knock-out (KO) mice treated with 0.2% cuprizone in feed for 5 weeks. Red arrow point to external capsule. Scale bars: 100 µm.

Figure S14. Quantitative image analysis of microglia/astrocyte numbers and morphology of stained Iba1 and GFAP brain sections. **a** Original image. **b** Valid somas are shown in red, **c** all microglia processes are marked green, **d** proximal processes (restricted by maximal distance from soma center, red circle) are shown in green, **e** distal processes (restricted by maximal distance from soma center, red circle) are marked green, **f** visible microglia for valid soma, restricted to circular reference region (red circle) defining “proximity” (for computation of integrated optical density) are marked green and **g** the final result for the image analysis shows soma (blue), with proximal (red) and all other processes (green).

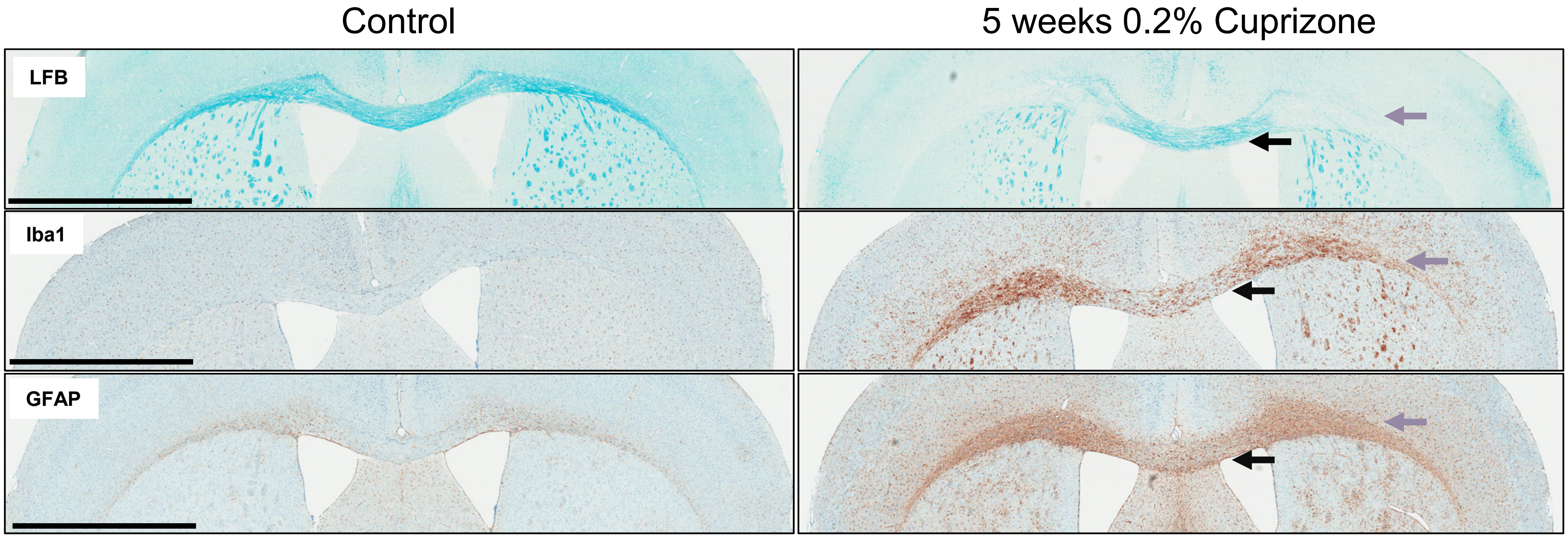

Supp. Fig. 1

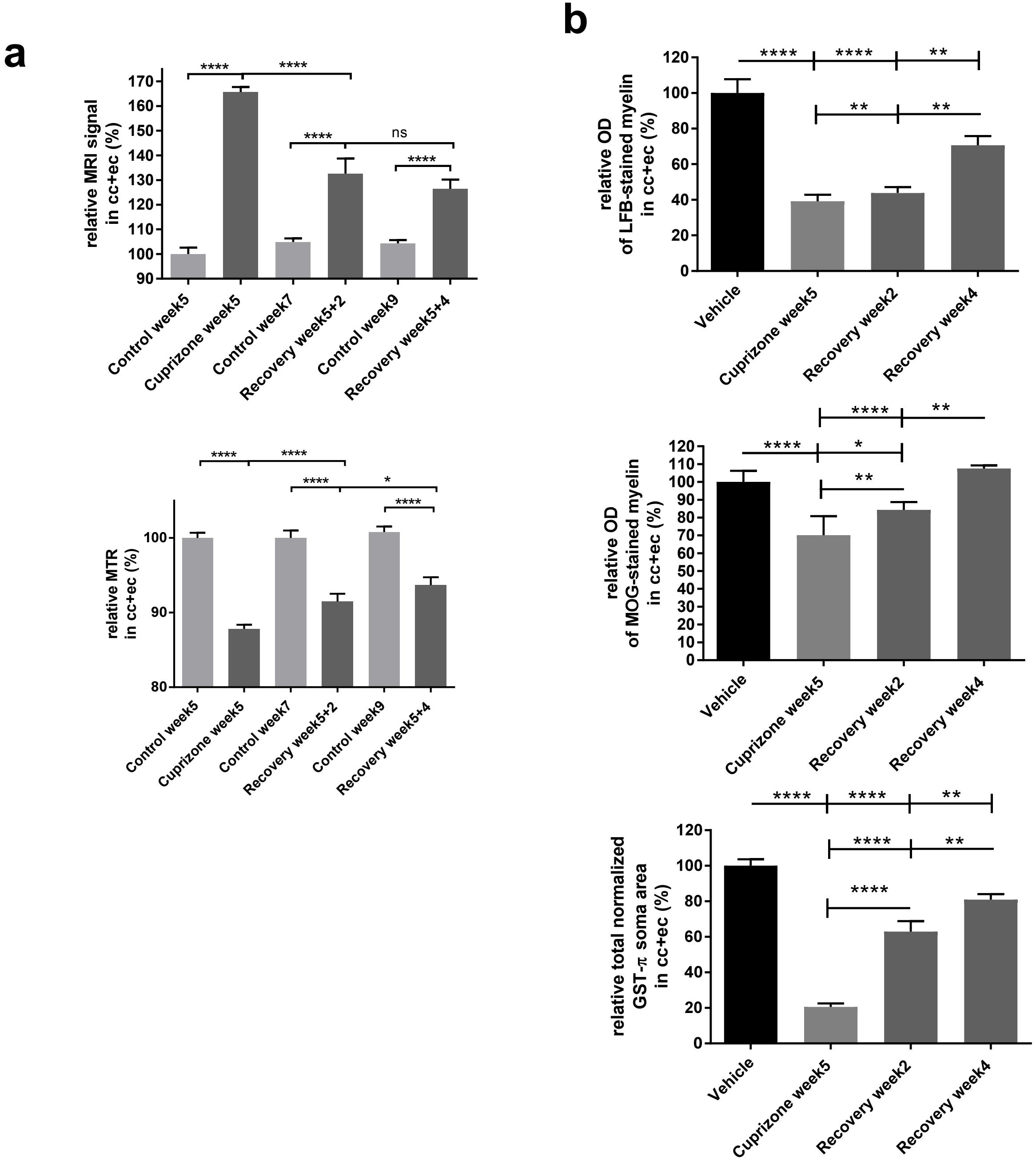

a
relative MRI signal in cc+ec (%)
Control week5
Cuprizone week5
Control week7
Recovery week5+2
Control week9
Recovery week5+4
ns
relative MTR in cc+ec (%)
b
relative OD of LFB-stained myelin in cc+ec (%)
Vehicle
Cuprizone week5
Recovery week2
Recovery week4
relative OD of MOG-stained myelin in cc+ec (%)
relative total normalized GST-π soma area in cc+ec (%)

Supp. Fig. 2

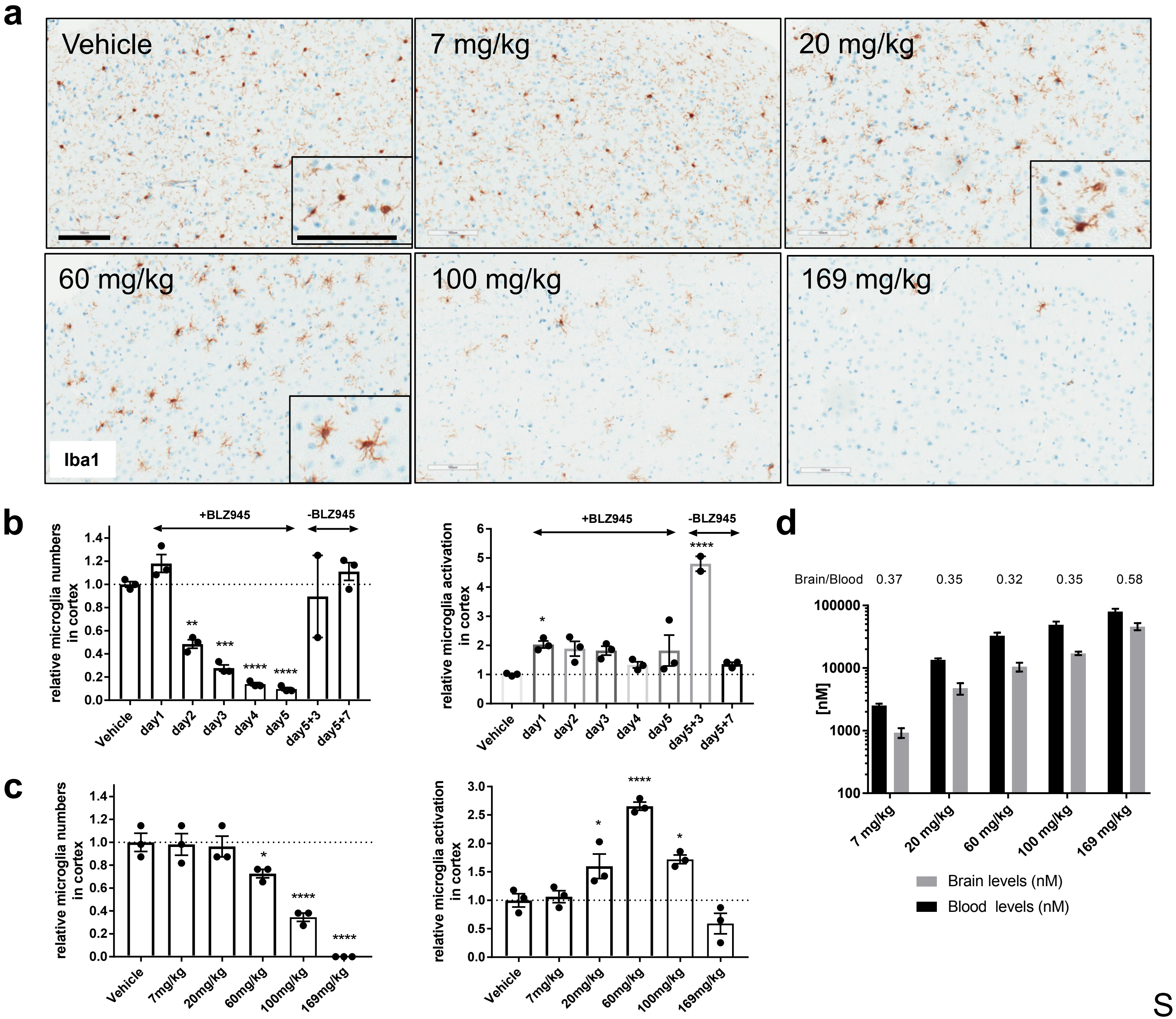
a
Vehicle
7 mg/kg
20 mg/kg
60 mg/kg
100 mg/kg
169 mg/kg
Iba1
b
+BLZ945
-BLZ945
relative microglia numbers in cortex
relative microglia activation in cortex
Vehicle
day1
day2
day3
day4
day5
day5+3
day5+7
c
7mg/kg
20mg/kg
60mg/kg
100mg/kg
169mg/kg
d
Brain/Blood 0.37 0.35 0.32 0.35 0.58
[nM]
Brain levels (nM)
Blood levels (nM)

Supp. Fig. 3

a

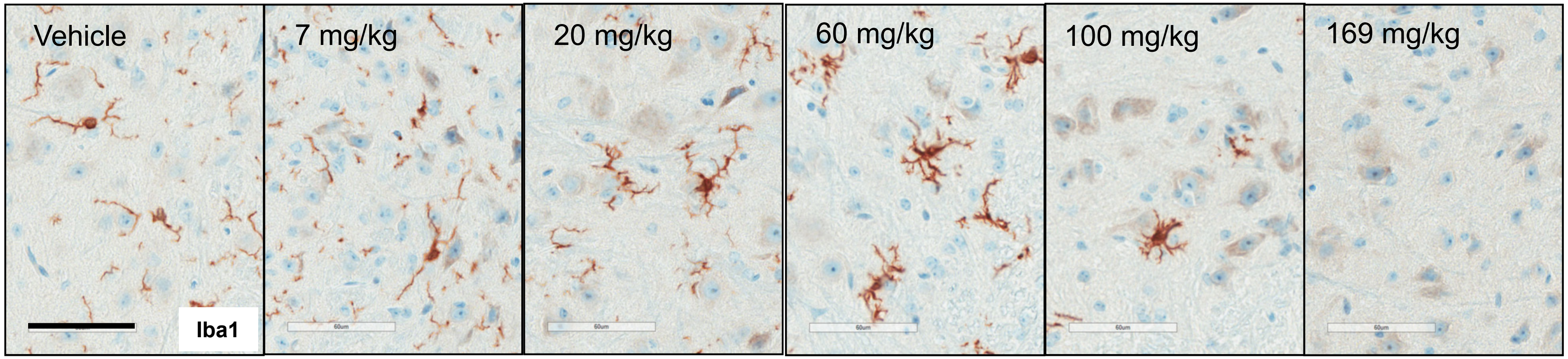

Vehicle
7 mg/kg
20 mg/kg
60 mg/kg
100 mg/kg
169 mg/kg
Iba1

b

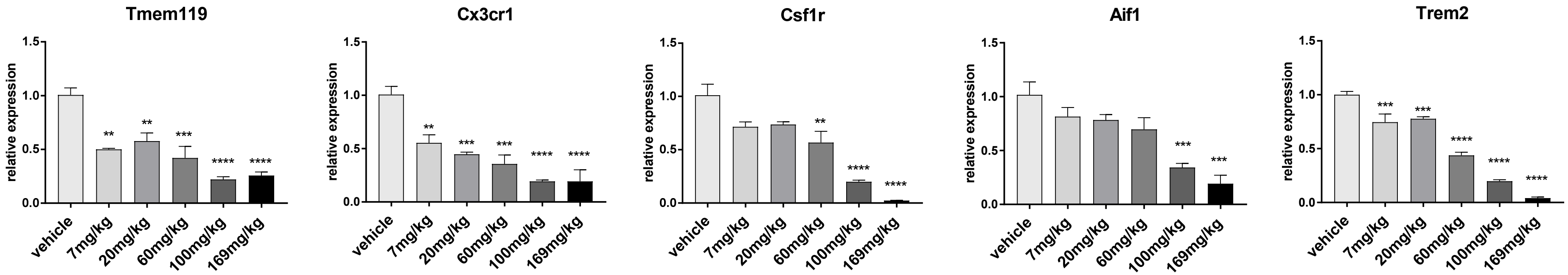

Tmem119
Cx3cr1
Csf1r
Aif1
Trem2
relative expression
1.5
1.0
0.5
0.0
vehicle
7mg/kg
20mg/kg
60mg/kg
100mg/kg
169mg/kg

Supp. Fig. 4

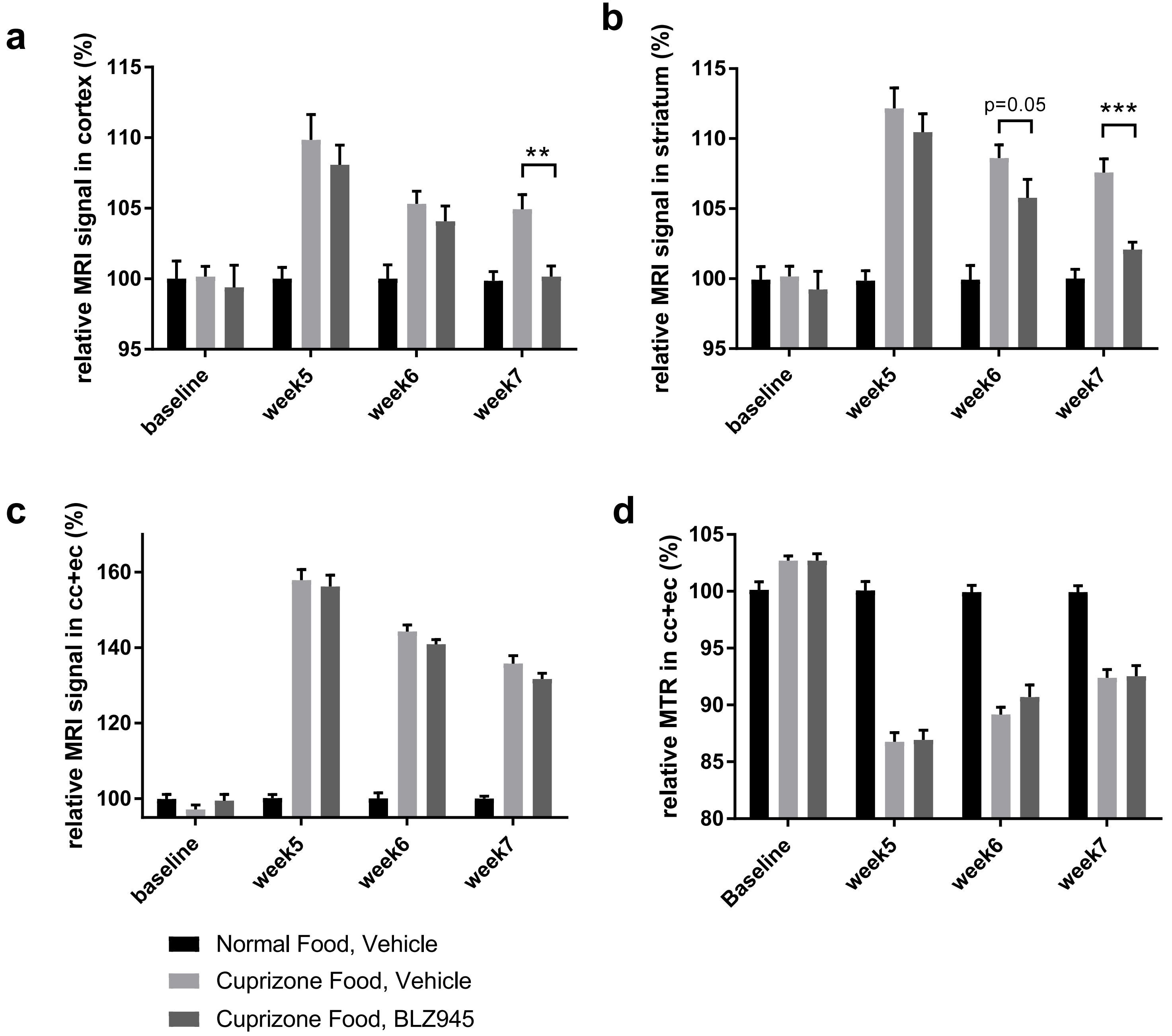
a
relative MRI signal in cortex (%)
115
110
105
100
95
**
baseline
week5
week6
week7
b
relative MRI signal in striatum (%)
115
110
105
100
95
p=0.05
***
baseline
week5
week6
week7
c
relative MRI signal in cc+ec (%)
160
140
120
100
baseline
week5
week6
week7
d
relative MTR in cc+ec (%)
105
100
95
90
85
80
Baseline
week5
week6
week7
Normal Food, Vehicle
Cuprizone Food, Vehicle
Cuprizone Food, BLZ945

Supp. Fig. 5

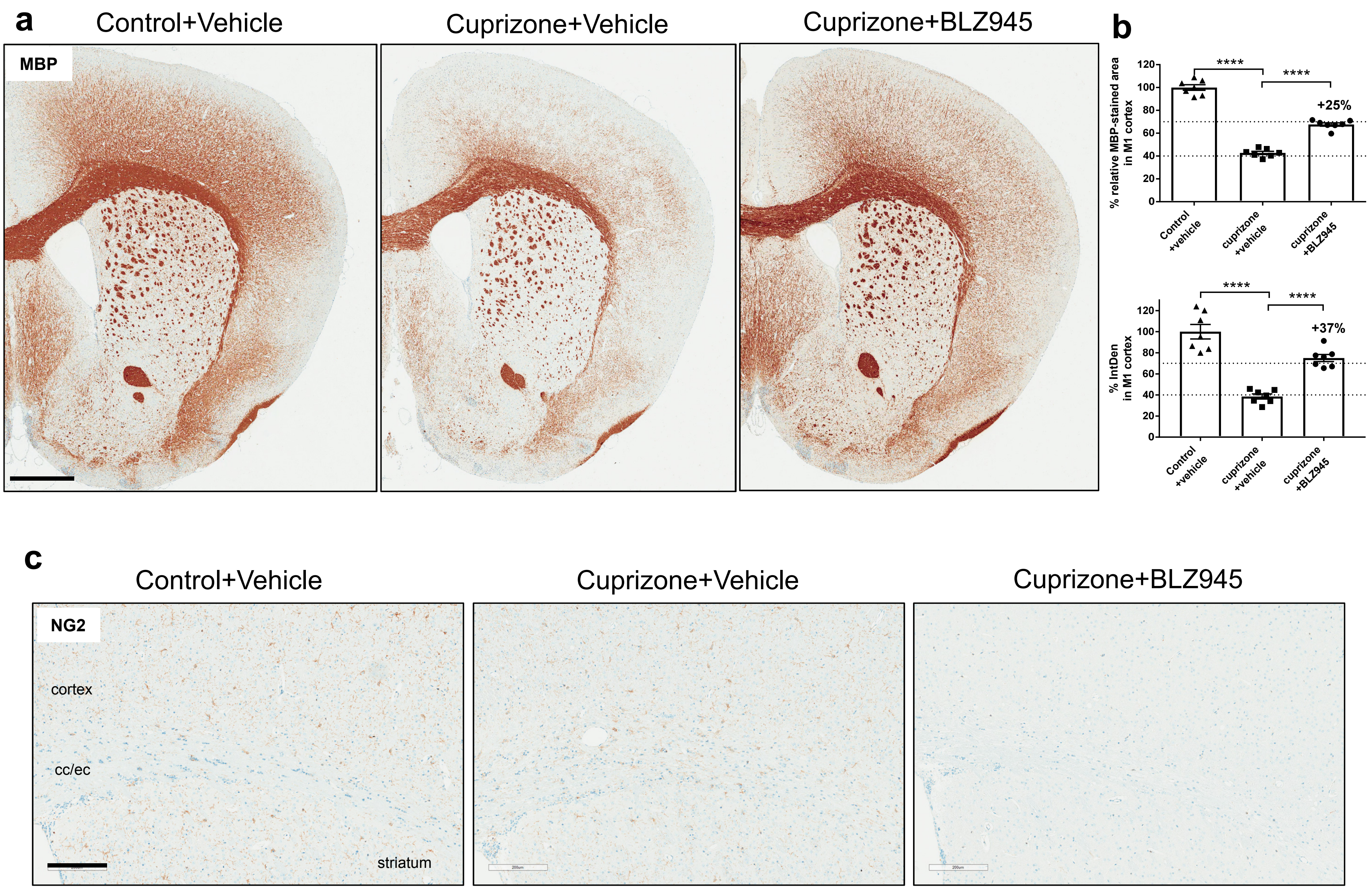
a
Control+Vehicle
Cuprizone+Vehicle
Cuprizone+BLZ945
MBP
b
% relative MBP-stained area in M1 cortex
****
****
+25%
Control +vehicle
cuprizone +vehicle
cuprizone +BLZ945
% IntDen in M1 cortex
+37%
c
NG2
cortex
cc/ec
striatum

Supp. Fig. 6

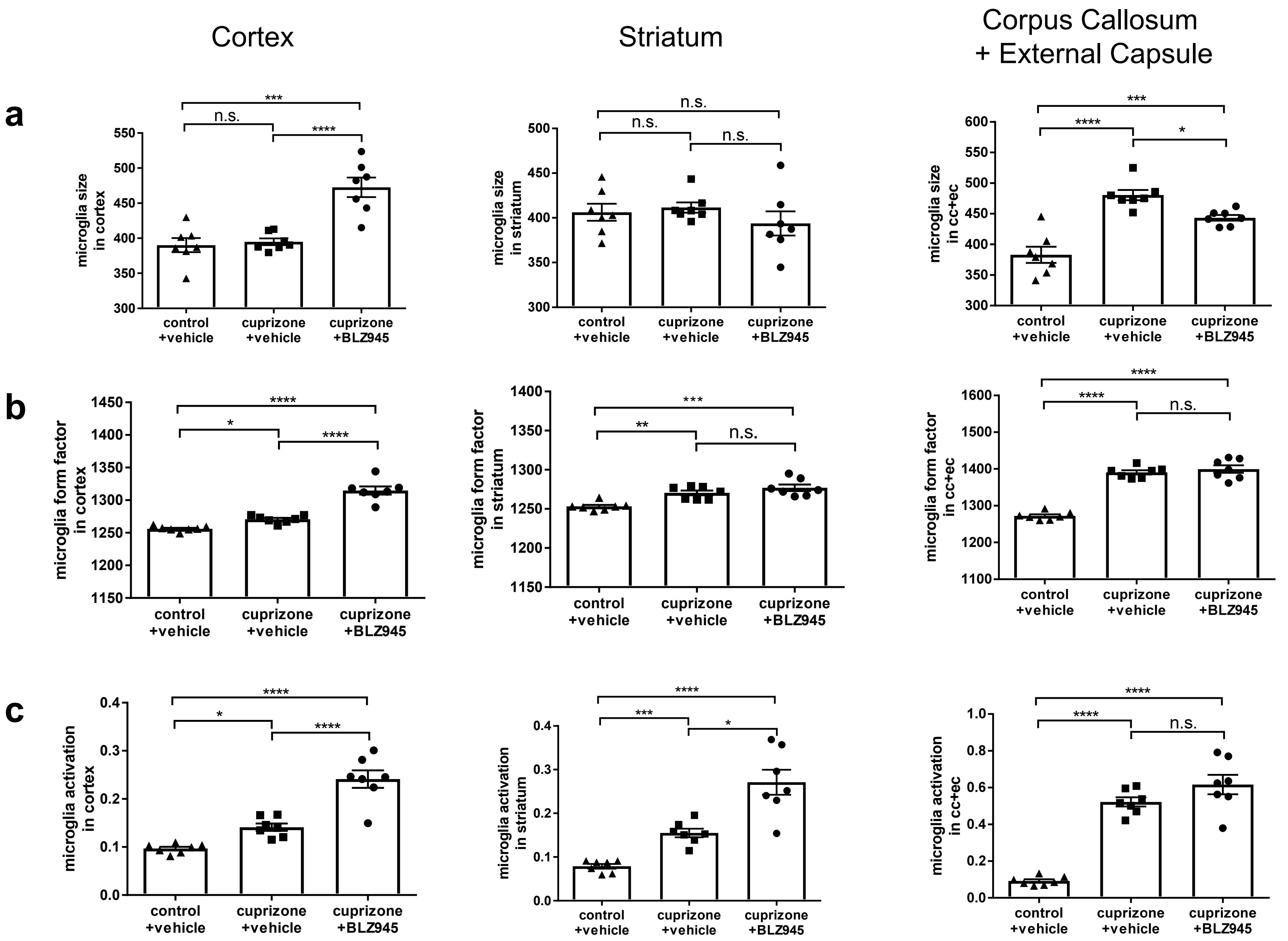
Cortex
Striatum
Corpus Callosum
+ External Capsule
a
b
c
microglia size in cortex
microglia size in striatum
microglia size in cc+ec
microglia form factor in cortex
microglia form factor in striatum
microglia form factor in cc+ec
microglia activation in cortex
microglia activation in striatum
microglia activation in cc+ec
control +vehicle
cuprizone +vehicle
cuprizone +BLZ945
n.s.
*
**
***
****

Supp. Fig. 7

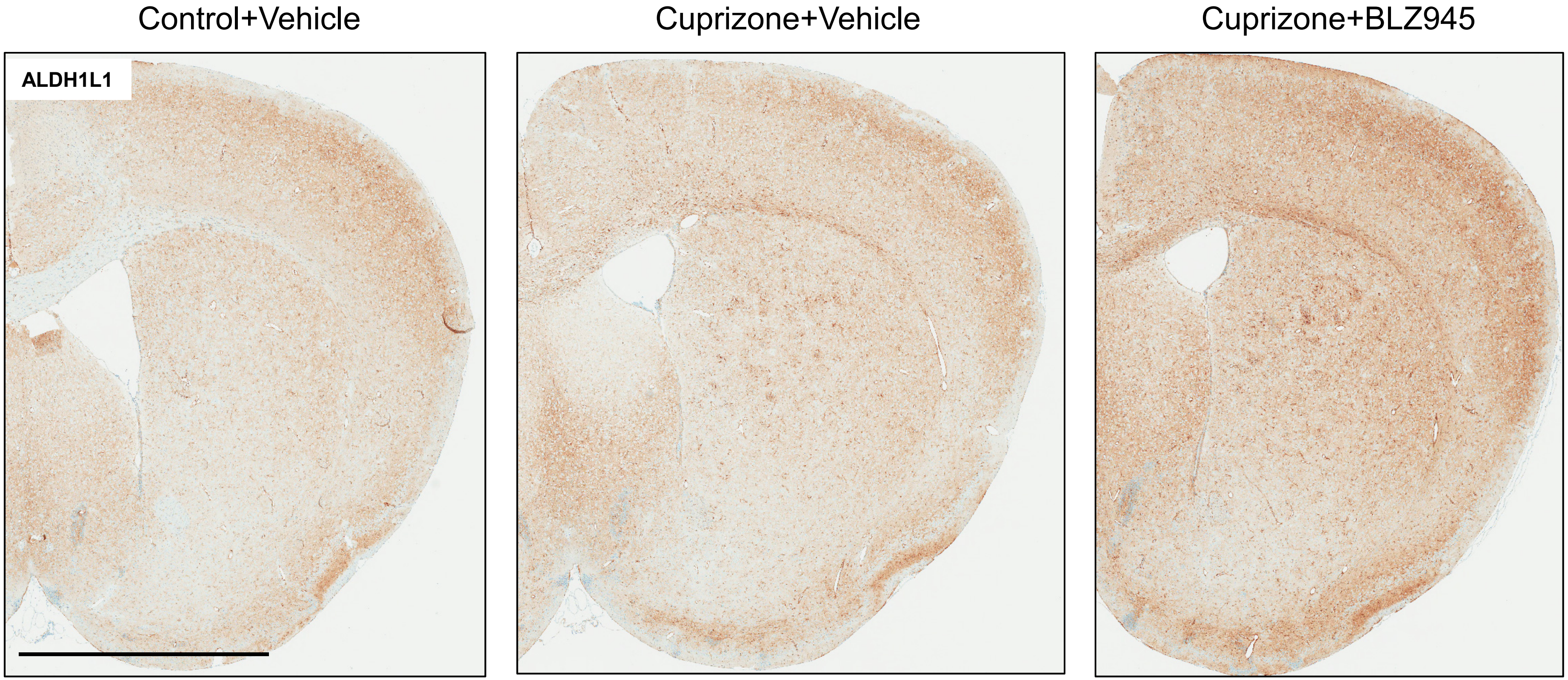
Control+Vehicle
Cuprizone+Vehicle
Cuprizone+BLZ945
ALDH1L1

Supp. Fig. 8

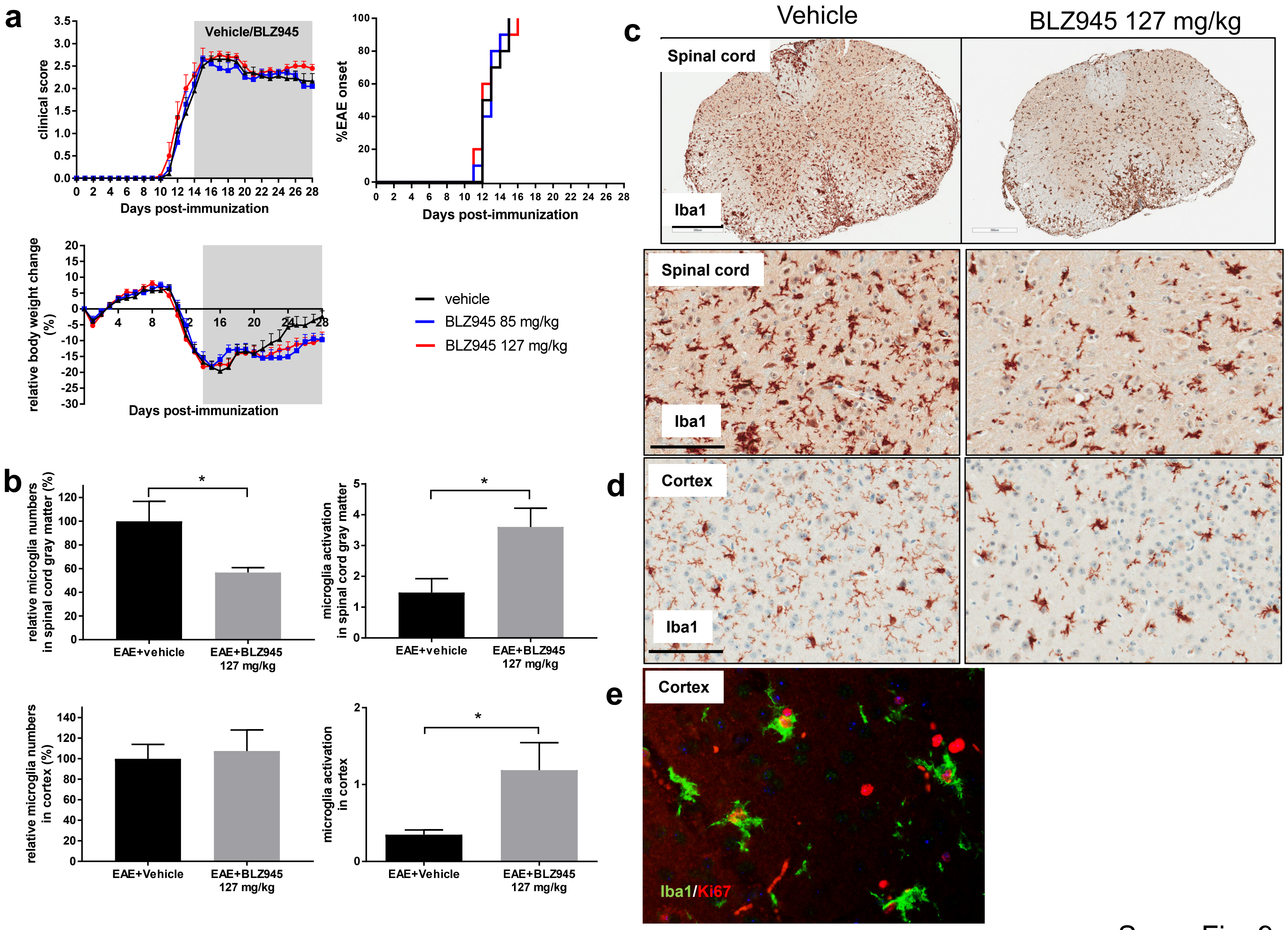

a
Vehicle/BLZ945
clinical score
Days post-immunization
%EAE onset
Days post-immunization
relative body weight change (%)
Days post-immunization
vehicle
BLZ945 85 mg/kg
BLZ945 127 mg/kg
b
relative microglia numbers in spinal cord gray matter (%)
EAE+vehicle
EAE+BLZ945 127 mg/kg
microglia activation in spinal cord gray matter
EAE+vehicle
EAE+BLZ945 127 mg/kg
relative microglia numbers in cortex (%)
EAE+Vehicle
EAE+BLZ945 127 mg/kg
microglia activation in cortex
EAE+Vehicle
EAE+BLZ945 127 mg/kg
c
Vehicle
BLZ945 127 mg/kg
Spinal cord
Iba1
Spinal cord
Iba1
d
Cortex
Iba1
e
Cortex
Iba1/Ki67

Supp. Fig. 9

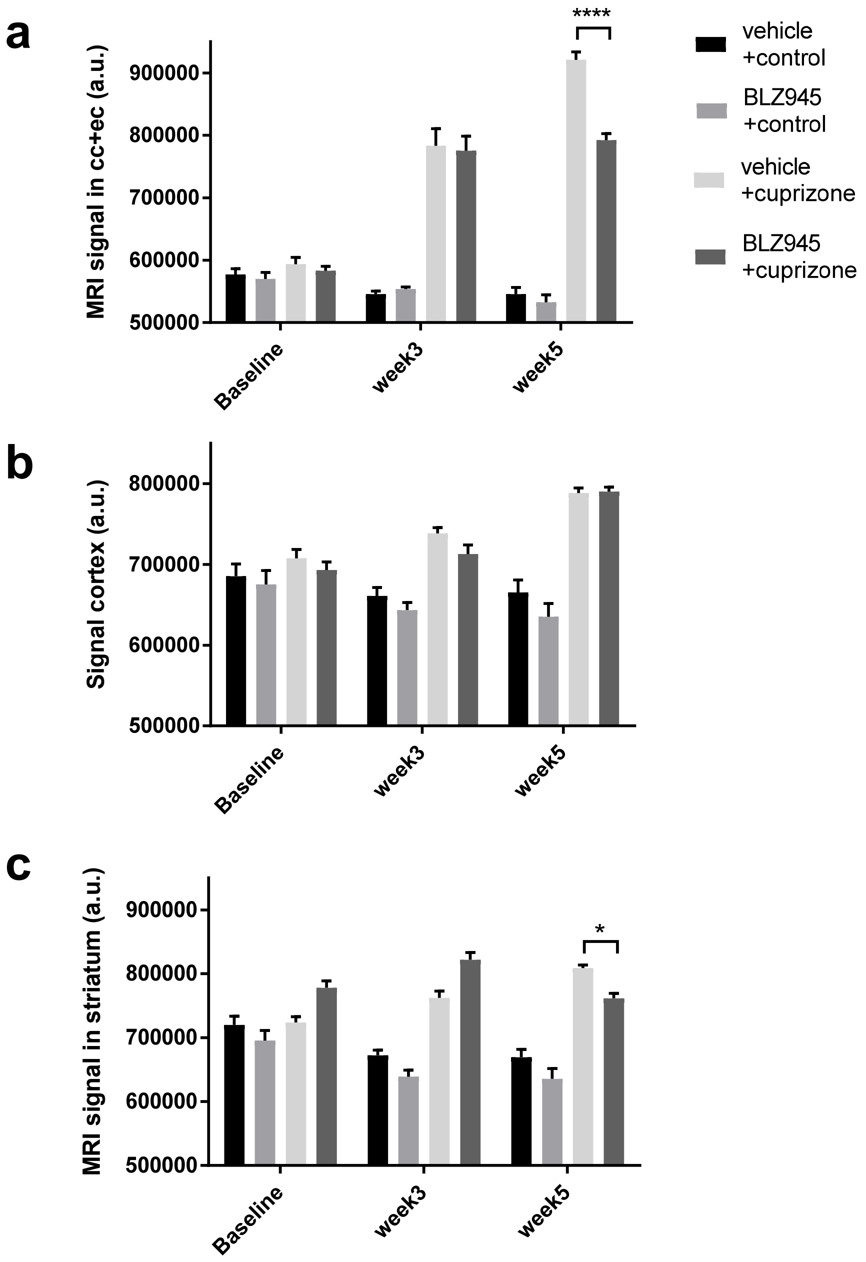
a
b
c
MRI signal in cc+ec (a.u.)
Signal cortex (a.u.)
MRI signal in striatum (a.u.)
900000
800000
700000
600000
500000
Baseline
week3
week5
****
*
vehicle +control
BLZ945 +control
vehicle +cuprizone
BLZ945 +cuprizone

Supp. Fig. 10

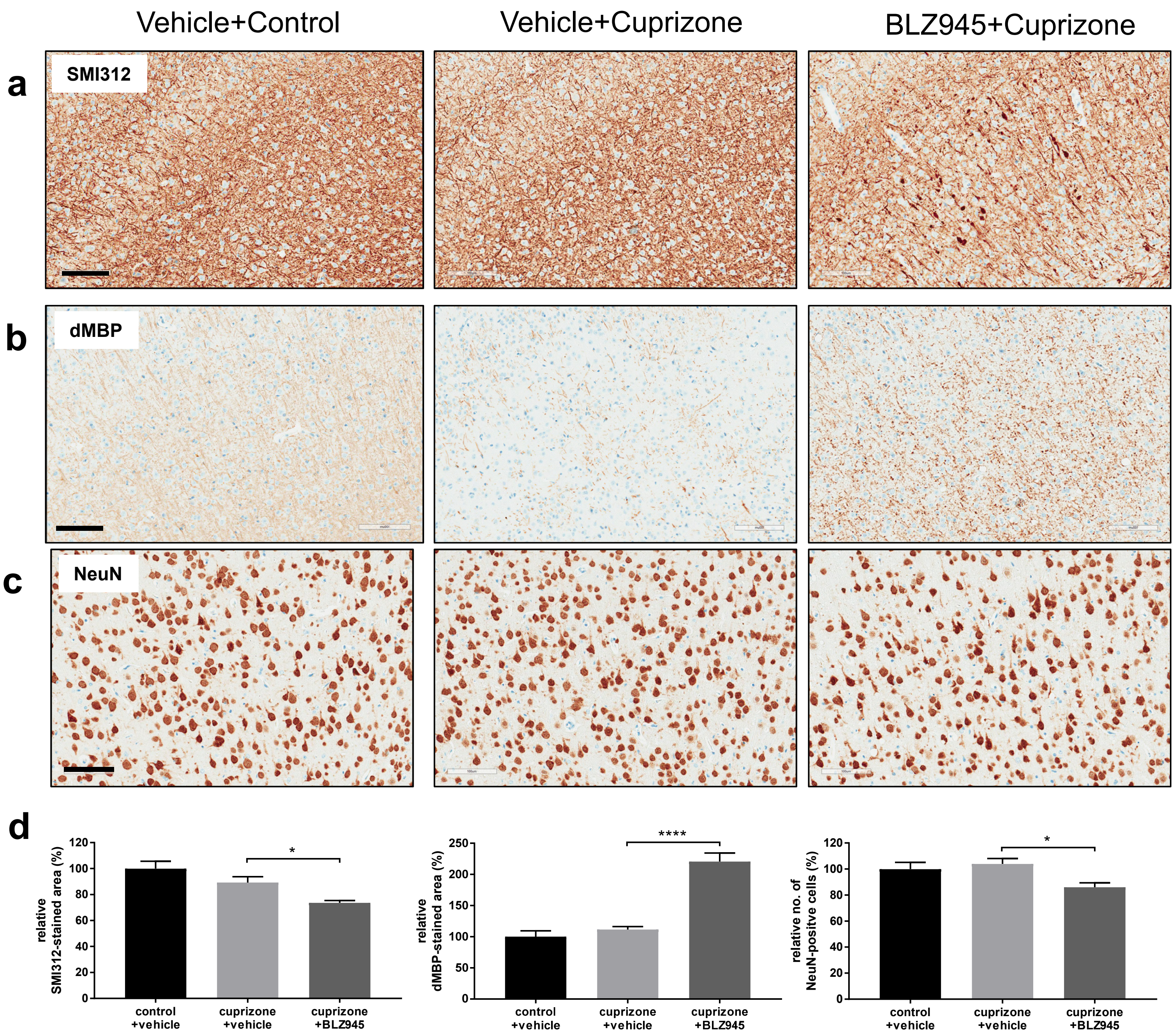
Vehicle+Control
Vehicle+Cuprizone
BLZ945+Cuprizone
a
SMI312
b
dMBP
c
NeuN
d
relative SMI312-stained area (%)
120
100
80
60
40
20
0
*
control +vehicle
cuprizone +vehicle
cuprizone +BLZ945
relative dMBP-stained area (%)
250
200
150
100
50
0
****
control +vehicle
cuprizone +vehicle
cuprizone +BLZ945
relative no. of NeuN-positive cells (%)
120
100
80
60
40
20
0
*
control +vehicle
cuprizone +vehicle
cuprizone +BLZ945

Supp. Fig. 11

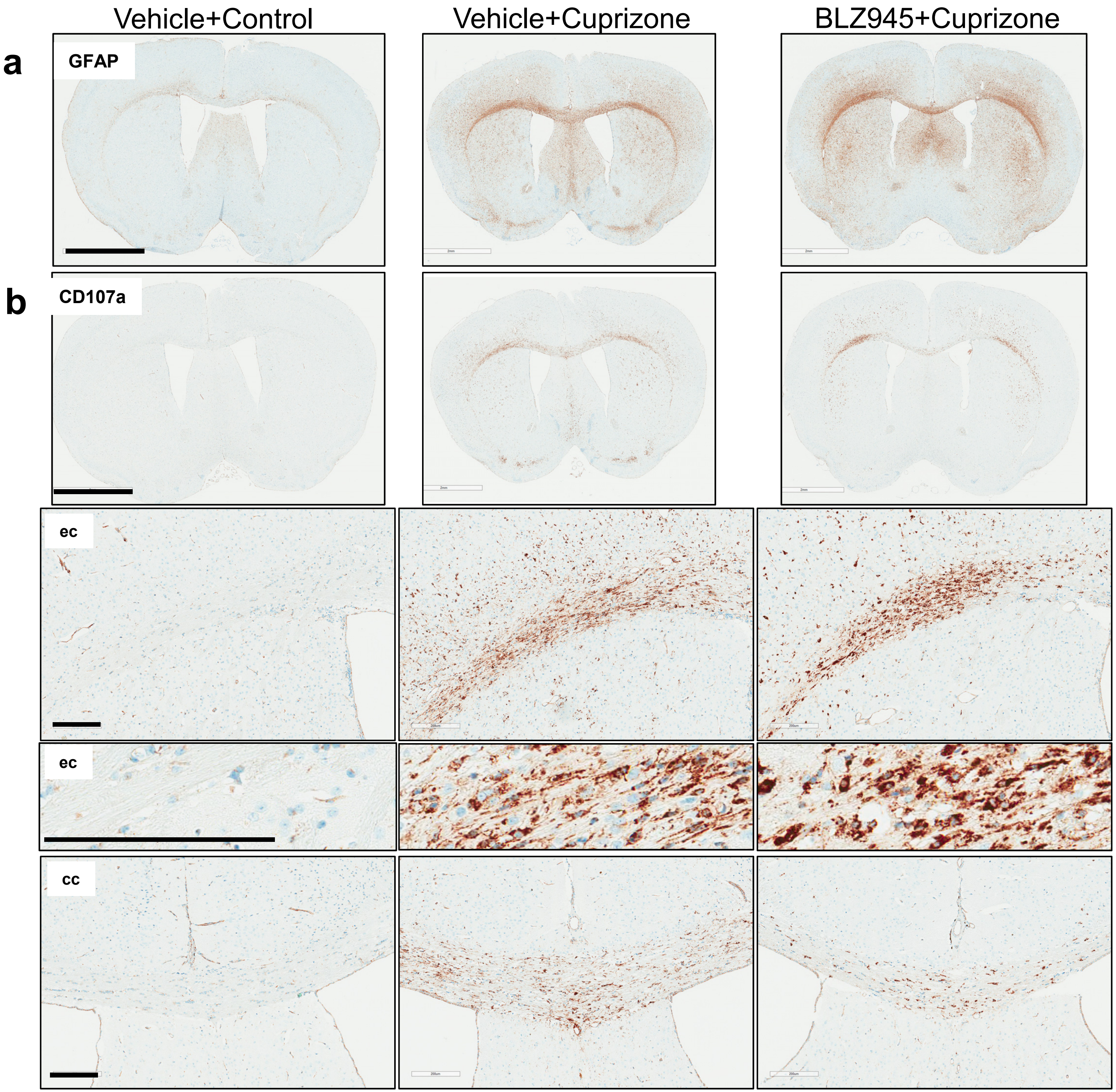
Vehicle+Control
Vehicle+Cuprizone
BLZ945+Cuprizone
a
GFAP
b
CD107a
ec
ec
cc

Supp. Fig. 12

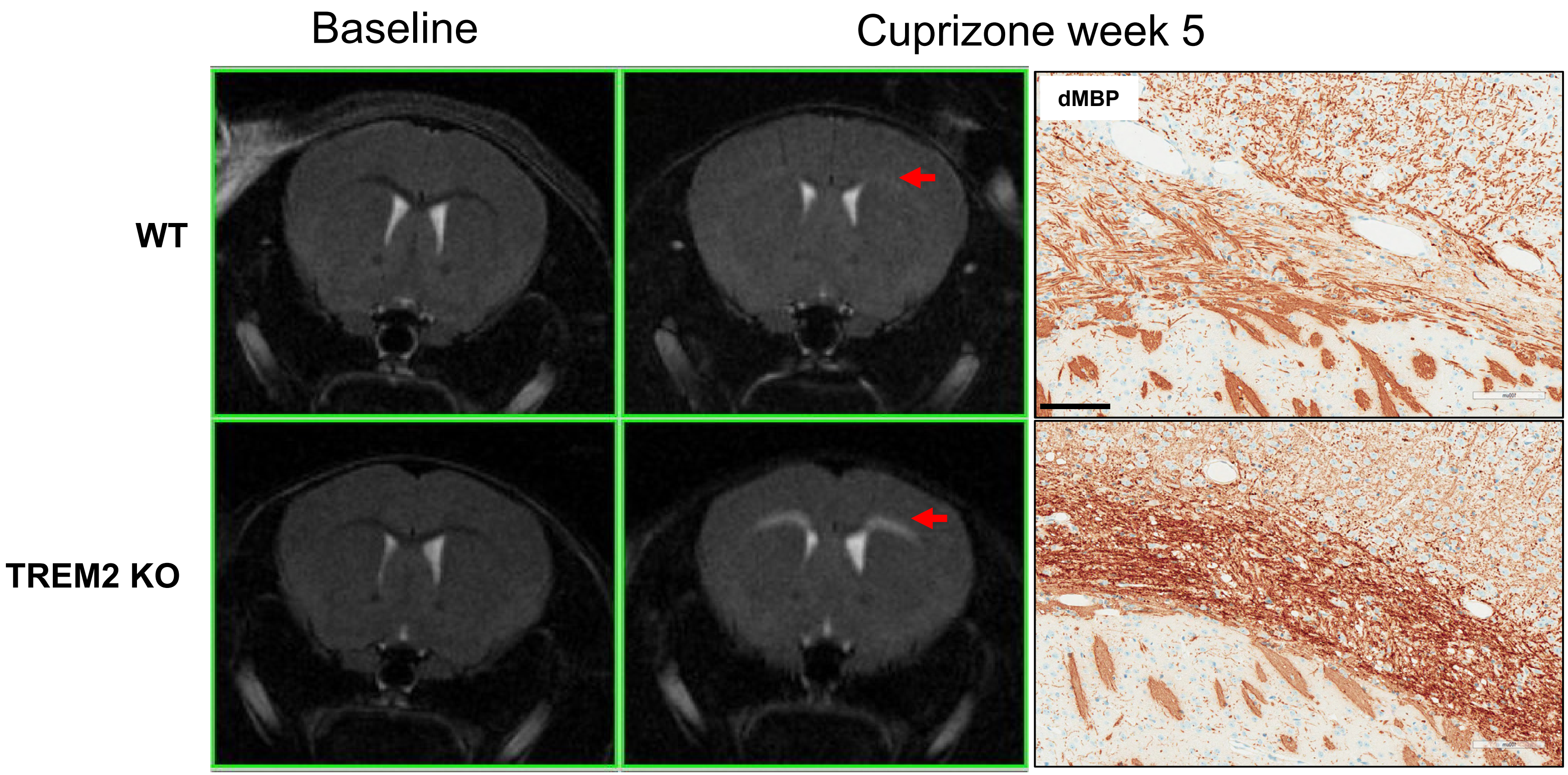

Supp. Fig. 13

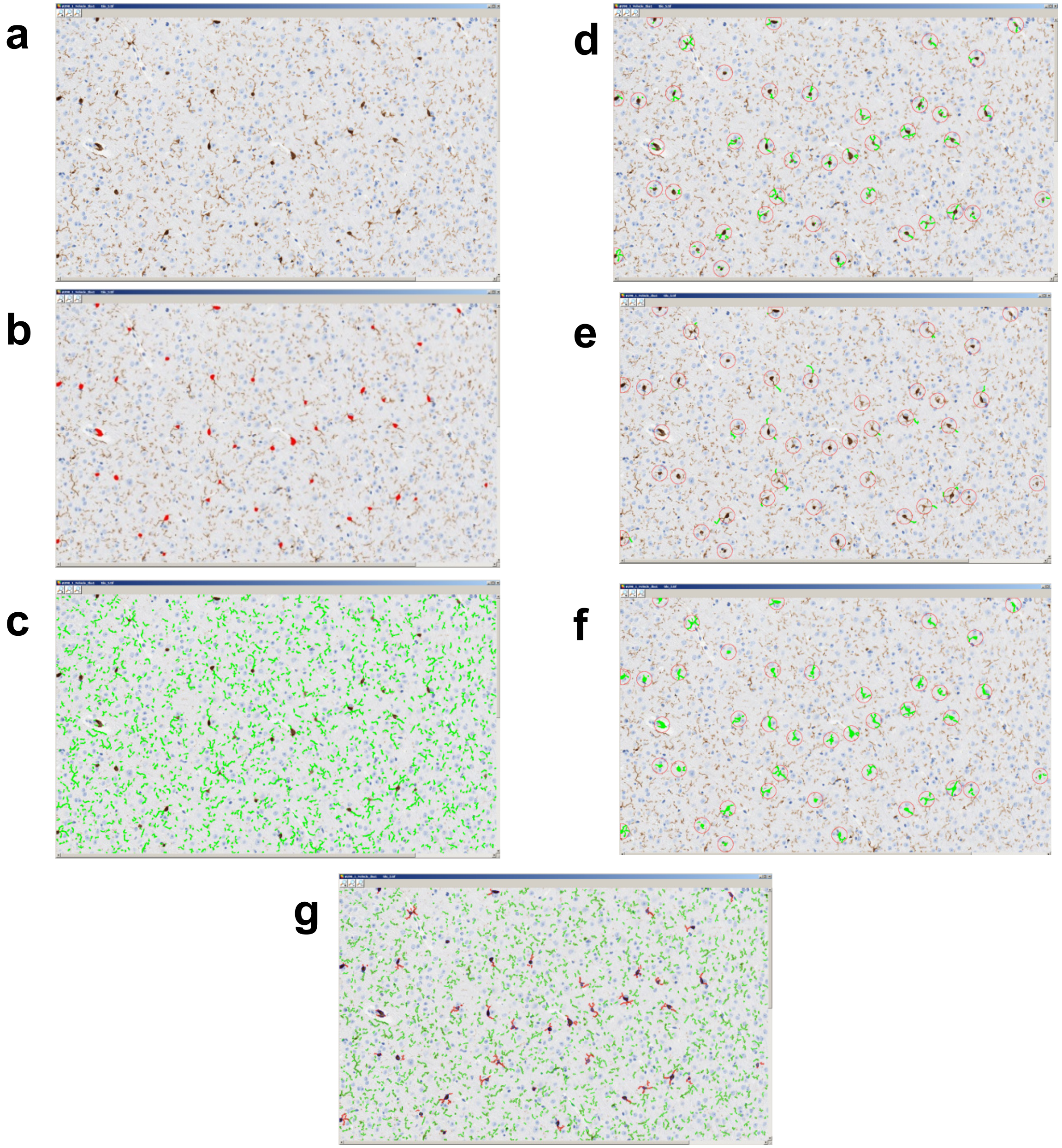

Supp. Fig. 14
